# Putative plasmid prophages of *Bacillus cereus sensu lato* may hold the key to undiscovered phage diversity

**DOI:** 10.1038/s41598-021-87111-3

**Published:** 2021-04-07

**Authors:** Emma G. Piligrimova, Olesya A. Kazantseva, Andrey N. Kazantsev, Nikita A. Nikulin, Anna V. Skorynina, Olga N. Koposova, Andrey M. Shadrin

**Affiliations:** 1 Laboratory of Bacteriophage Biology, G.K. Skryabin Institute of Biochemistry and Physiology of Microorganisms, Pushchino Scientific Center for Biological Research of the Russian Academy of Sciences, Federal Research Center, 142290 Pushchino, Russia; 2 P. N. Lebedev Physical Institute of the Russian Academy of Sciences, Pushchino Radio Astronomy Observatory, Pushchino, 142290 Russia

**Keywords:** Bacteriophages, Bacterial genetics

## Abstract

Bacteriophages are bacterial viruses and the most abundant biological entities on Earth. Temperate bacteriophages can form prophages stably maintained in the host population: they either integrate into the host genome or replicate as plasmids in the host cytoplasm. As shown, tailed temperate bacteriophages may form circular plasmid prophages in many bacterial species of the taxa *Firmicutes*, *Gammaproteobacteria* and *Spirochaetes*. The actual number of such prophages is thought to be underestimated for two main reasons: first, in bacterial whole genome-sequencing assemblies, they are difficult to distinguish from actual plasmids; second, there is an absence of experimental studies which are vital to confirm their existence. In *Firmicutes*, such prophages appear to be especially numerous. In the present study, we identified 23 genomes from species of the *Bacillus cereus* group that were deposited in GenBank as plasmids and may belong to plasmid prophages with little or no homology to known viruses. We consider these putative prophages worth experimental assays since it will broaden our knowledge of phage diversity and suggest that more attention be paid to such molecules in all bacterial sequencing projects as this will help in identifying previously unknown phages.

## Introduction

Bacteriophages (phages) are viruses that infect bacteria. Bacteriophages have developed fundamentally different lifecycle strategies: virulent phages start replicating their DNA and assembling virion particles shortly after entering the host cell (lytic cycle), while temperate phages can either enter the lytic or lysogenic cycle. The lysogenic cycle means the phage DNA is integrated into the host genome (integrated prophages) or replicates as a circular or linear plasmid in the host cytoplasm (plasmid prophages). Prophages can be maintained in the host population for a long time and then switch to the lytic cycle (prophage induction). Biologically active prophages (i.e. those still inducible and able to produce functional phage particles) can degenerate into inactive prophage remnants, unable to ensure the production of functional phage particles due to mutations in key genes.

There have been many reports describing temperate tailed dsDNA bacteriophages replicating as circular plasmids in the prophage state instead of integrating their genomes into the host DNA. This phenomenon was first noticed long before the Next generation sequencing (NGS) era when a number of phages were experimentally demonstrated to form plasmid prophages, including SU-11^1^ and phi20^2^ whose entire genome sequences have still not been fully documented. *Myoviridae* prophage P1 of the *E. coli* K12 was the first circular plasmid prophage to be discovered^3^ and later became an object model in molecular biology along with bacteriophages lambda and T4. Its replication as a low-copy number plasmid has been thoroughly investigated^4^ and key proteins of this process have been fully identified.

As genome sequencing services continue to be more and more accessible such phages are reported more often and this phenomenon continues to gain more recognition as a real variant of the lysogenic cycle apart from the well-known classical scenario, which fundamentally implies the integration of phage genetic material into the host DNA. Several phages with experimentally confirmed plasmid prophages are listed in Table 1. Although isolated from evolutionary remote bacteria, they have conserved characteristics enabling extrachromosomal replication, including proteins that have long been known in bacteria as participating in plasmid segregation and in the resolution of chromosomal and plasmid dimers after replication.

Examples of such proteins are two related site-specific tyrosine recombinases, XerC and XerD, encoded by most bacteria where they serve to resolve dimers of circular chromosomes and plasmids to ensure proper distribution of the genetic material to daughter cells^5^. Most phages that form circular plasmid prophages possess one or two tyrosine recombinases related to XerCD, which are believed to participate in resolving dimeric forms of the phage DNA after replication (Table 1). However, site-specific tyrosine recombinases can serve other functions as well, most notably the integration and excision of mobile genetic elements. For example, $\lambda$ integrase, the founding member of the tyrosine recombinase protein family, enables the integration of the bacteriophage lambda DNA into the *E. coli* chromosome^5^. Even the XerC and XerD recombinases act like integrases under certain conditions, as has been shown for the *V. cholera* filamentous bacteriophage CTX$\varphi$ (order *Tubulavirales*, family *Inoviridae*) which recruits the XerC and XerD recombinases of its host for integration^6^. Another unrelated family of site-specific recombinases includes serine recombinases, which like site-specific tyrosine recombinases are also not unusual in phage genomes and can function as resolvases, invertases, transposases and integrases. However, unlike tyrosine recombinases, in the case of serine recombinases, we can, to some degree, distinguish between their functions by estimating their length and domain arrangement^7,8^. Thus, the presence of tyrosine and serine recombinases is not a diagnostic feature of bacteriophages with circular plasmid prophages, but something that may allow for a deeper understanding of their lifecycles.

**Table 1:** Some phages with experimentally proven circular plasmid prophages.

Phage	Publication year	Genome size, bp	DNA packaging mechanism	Partitioning proteins	Recombinases	Morphotype	Host	GenBank accession number	References
*Enterobacteria* phage P1	1951	94,800	Headful	ParA-Ia-type ATPase; ParB	Site-specific tyrosine recombinase Cre	M	*E. coli*	NC_005856.1	^3,9^
*Clostridium* virus c-st	2005	185,683	404-bp DTRs	TubZ; TubR	One Xer family site-specific tyrosine recombinase	S	*C. botulinum*	AP008983.1	^10,11^
*Bacillus* phage IEBH	2011	53,104	Headful	ParM-like protein; RHH domain-containing protein	–	S	*B. cereus*	NC_011167.1	^12^
*Clostridium* phage phiCD38-2	2011	41,090	Headful	ParA-Ib-type ATPase; ParB-III	One site-specific tyrosine recombinase resembling Xer family recombinases; Small serine recombinase of the Resolvase and Invertase subfamily	S	*C. difficile*	NC_015568.1	^13^
*Vibrio* phage pVv01	2014	79,263	Probably headful	ParA-Ib-type ATPase; RHH domain-containing protein	Three site-specific tyrosine recombinases similar to Xer family recombinases	M	*V. vulnificus*	HG803186.1	^14^
*Bacillus* phage Bp8p-C	2015	151,417	Supposedly LTRs (by the similarity to the *Bacillus* phage phiAGATE)	ParM-like protein; HTH domain-containing adapter protein	–	M	*B. pumilus*	KJ010547.1	^15^
*Enterobacteria* phage D6	2018	91,159	Not determined	ParA-Ib-type ATPase; RHH domain-containing protein	One site-specific tyrosine recombinase resembling P1 Cre recombinase; Small serine recombinase of the resolvase and invertase subfamily	M	*E. coli*	NC_050154.1	^16^
*Bacillus* phage vB_BtS_B83	2019	49,952	Headful	ParM-like protein; RHH domain-containing protein	Two Xer family site-specific tyrosine recombinases	S	*B. thuringiensis*	NC_048762.1	^17^

*DTRs:* direct terminal repeats, *LTRs:* long terminal repeats, *RHH:* ribbon-helix-helix, *HTH:* helix-turn-helix, *M:* Myoviridae, *S:* Siphoviridae.

The same conclusion may be reached with regard to plasmid partitioning proteins, another group of proteins highly conserved in phages replicating extrachromosomally as circular plasmids. There are three major classes of plasmid partitioning systems, each includes three components: a nucleotide-driven motor protein, a small DNA-binding adapter protein encoded immediately downstream of the motor protein and a centromere-like DNA region to which the adaptor binds^18^. The motor proteins move adapter-bound newly replicated plasmid molecules to the opposite cell poles to ensure that both daughter cells inherit an equal number of plasmids. The motor protein defines the class of the partitioning system: ParA-like Walker A-type ATPase (class I), ParM-like actin-like protein (class II) or TubZ-like tubulin-like protein (class III), the first two being the most prevalent. ParA proteins are classified into ParA-Ia family ATPases containing an additional N-terminal helix-turn-helix (HTH) domain, and ParA-Ib family ATPases that do not contain HTH domains and bind non-specifically to DNA^19^. Adapter proteins working together with ParA ATPases are in turn divided into ParB-I and ParB-II subfamilies recognizing centromere DNA via an HTH domain as well as ParB-III subfamily recognizing the centromere region via a ribbon-helix-helix (RHH) motif^19^.

Plasmid prophages are very likely to possess one or the other type of partitioning systems (Table 1). However, the presence of such proteins alone does not guarantee that the phage replicates as a plasmid. Several phage actin-like and tubulin-like proteins have been shown to participate not only in plasmid prophage segregation prior to cell division but also in intracellular transport of prophage molecules, e.g. to the cell membrane for replication and assembly of phage particles. An example of this includes the prophage CGP3 from *C. glutamicum* ATCC 13032 which encodes the actin-like protein AlpC and the adapter protein AlpA^20^, however in the host population the prophage is mostly integrated into the host chromosome. It can be spontaneously induced and excised from the host DNA, which results in phage DNA circularization and the expression of phage genes, including those coding for AlpC and AlpA, which, working together, direct the phage DNA molecules to the cell membrane, where additional replication occurs^20^. Thus, identifying a plasmid partitioning system encoded in a phage genome does not necessarily indicate that the phage has a plasmid prophage stage.

Supplementary to the two points covered above, even obtaining a circular phage-related contig in a bacterial whole-genome sequencing (WGS) assembly can not confirm, without unambiguity, that the prophage indeed exists as a circular plasmid. Circular phage contigs can in fact be obtained from linear phage DNA that contaminates sequencing libraries due to repeated sequences at the termini of phage chromosomes, which lead to overlapping reads closing the contigs into circles^21–23^. The same is possible in the case of *cos*-phages if their single-stranded terminal overhangs anneal during sequencing library preparation^22^.

In our previous work we isolated and described a novel *Bacillus*-infecting bacteriophage, vB_BtS_B83 (abbreviated as B83), which was originally deposited in GenBank and referred to as a plasmid^17,24^. B83 exists as a circular plasmid in the host cytoplasm and possesses a plasmid partitioning system and two genes coding for Xer recombinases. Since this discovery, we have grown interested in uncovering a real approximate number of such phages, the number of which we believe to be underestimated. The same issue was raised in 2018, when Eddie B. Gilcrease and Sherwood R. Casjens used the major capsid protein sequence of the *Enterobacteria* phage D6, distantly related to P1, to find putative D6-like prophages in the NCBI bacterial sequence database^16^. The authors came to the conclusion that the putative D6-like circular plasmid prophages are quite common in *Enterobacteriales* but rarely annotated as being phage-related. They also noted that more attention should be paid to such molecules in all bacterial sequencing projects as such prophages are likely to be common in many bacterial phyla^16^.

In the present study, we analyze plasmid genomes from species of the *Bacillus cereus* group for the presence of key proteins which are characteristic of plasmid prophages and we identify genomes (putative plasmid prophages) which may very likely belong to plasmid prophages. Among the identified putative plasmid prophages, 23 are especially interesting for they show little or no similarity to known viruses.

## Methods

### Database of plasmid proteins

Eighteen *B. cereus sensu lato* species taxonomically validated by the end of March 2019^25^,were used for the analyzes. The species were: *B. cereus*, *B. anthracis*, *B. thuringiensis*, *B. cytotoxicus*, *B. albus*, *B. luti*, *B. mobilis*, *B. nitratireducens*, *B. pacificus*, *B. paramycoides*, *B. proteolyticus*, *B. pseudomycoides*, *B. toyonensis*, *B. tropicus*, *B. weihenstephanensis*, *B. wiedmannii*, *B. mycoides* and *B. parantracis*. For each species, all complete plasmid genomes, including circular WGS contigs, were found in the NCBI INSDC (GenBank) database using the query ‘*Bacillus* $^\star$species_name$^\star$ ‘[Organism]’ and filters: ‘genetic compartment: plasmid’, ‘sequence length: 15–500 kbp’. All the translated coding sequences (CDSs) of the selected plasmids were downloaded as protein multifasta files, one file for each species. To estimate the number of unique genomes, the complete nucleotide sequences of the plasmids were downloaded as a multifasta file and clustered using the MMseqs2 software with the parameters: –min-seq-id 0.99, -c 0.99.

### HMM profile creation and database searching

All the protein sequences found in the NCBI Protein non-redundant database using the query ‘phage AND terminase [Protein name]’ for each of the seven selected proteins: terminase, major capsid protein, tape measure protein, XerC, ParM, ParA and TubZ, were downloaded as multifasta files (one file for each query) and clustered using MMseqs2^26^ with the parameters: coverage 80%, min identity 20%.

For clusters with 10 and more sequences, multiple alignments were created with Clustal Omega^27^ and then used to build hidden Markov model profiles (HMMs) with the hmmbuild tool from the HMMer 3.2.1 package^28^. The resulted HMM profiles were used to search the pregenerated plasmid protein database for plasmid genomes with matches for at least terminase and major capsid protein models using the hmmsearch tool (E-value cutoff: from $10^{-1}$ to $10^{-3}$, database size: from 324 to 43,116 for different species). Due to a rather small number of plasmids, no HMM filtration was performed before the search, as all the false-positive matches caused by incorrect GenBank annotation of the proteins used to build HMMs could be easily detected during further manual reannotation.

### Analysis of selected genomes

All the plasmid genomes selected on the previous step were reannotated using RASTtk^29^, followed by functional annotation using an in-house python script enabling each CDS to be analysed with BLAST tools^30^ and HHpred^31^. The genomes were then manually shifted setting the predicted small terminase subunit genes as the starting points of the genomes. Next, homologous gene clusters from the predicted proteomes of the plasmids were computed using the get_homologues.pl script, part of the GET_HOMOLOGUES software 3.2.1^32^ with the COGtriangles algorithm^33,34^ (with a threshold of 75% for query coverage and $1 \times 10^{-5}$ for E-value on the all-against-all BLASTP results). Next, the compare_clusters.pl script from the same package was used to produce the pangenome matrix showing whether each of the genomes possesses a representative of each protein cluster. The resulted pangenome matrix was uploaded to Cytoscape 3.8.0^35^ and visualized as a bipartite protein-sharing network, applying the Prefuse Force Directed Layout algorithm and the default edge weight settings. The selected genomes were divided into groups based on shared protein content and various genomic characteristics such as genome length, number of protein-coding genes, number of tRNAs, key genes, BLASTN whole-genome identity and whole-genome synteny. The TBLASTX genome comparison diagrams were visualized with EasyFig 2.2.2^36^.

## Results

506 complete plasmid genomes from *B. cereus sensu lato* species were initially selected based on the genome size (15–500 kbp), and all the translated coding sequences (CDSs) of the selected plasmids were downloaded as multifasta files, one file for each species. To estimate the number of unique genomes, complete nucleotide sequences of all the selected plasmids were downloaded and clustered with MMseqs2, and 474 genomes out of the 506 have proven to be unique.

**Figure 1:**
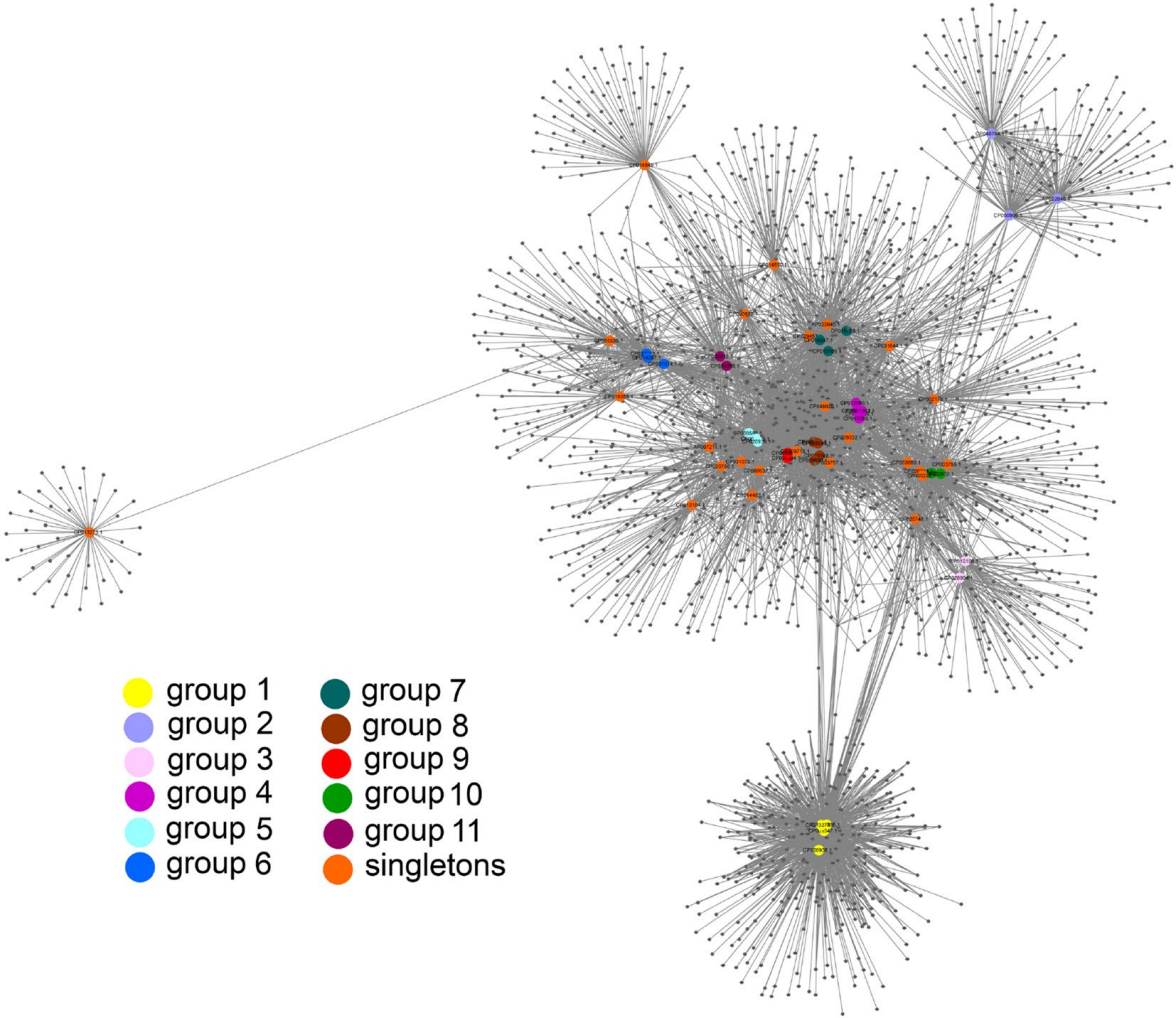
Protein-sharing network for 58 plasmid genomes from the *B. cereus* group species. Individual genomes are depicted as color circles, and protein clusters are depicted as dots. A line is drawn connecting a genome with a protein cluster if the genome harbors a representative of the protein cluster. The more closely genomes are located to each other, the more proteins they share. The network representation was produced using Cytoscape 3.8.0 (https://cytoscape.org).

The HMM profiles for seven proteins (see “Methods”) were used to search the multifasta files to find genomes possessing at least terminase and major capsid protein genes. This resulted in the selection of only 58 genomes including 17 plasmids from *B. cereus*, 33 plasmids from *B. thuringiensis*, one from *B. anthracis*, one from *B. tropicus*, two from *B. weihenstephanensis* and four from *B. mycoides*. Out of these 58 genomes, 33 were divided into 11 groups based on shared protein content and several genomic characteristics listed in “Methods”, leaving 25 singletons defined as those genomes that were not grouped with any others (Fig. 1).

### Group 1 (pBtic235-like)

**Figure 2:**
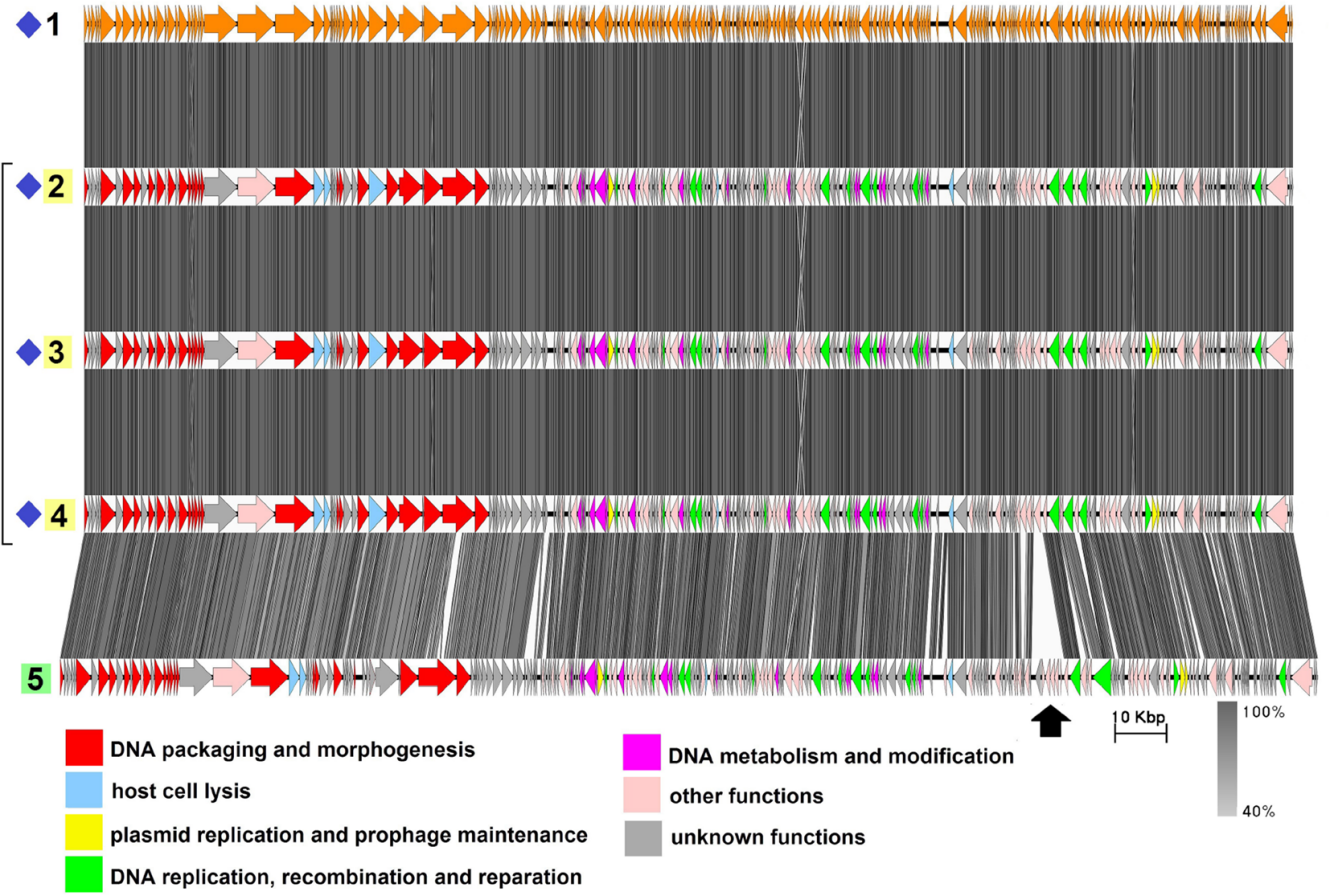
The TBLASTX genome comparison performed and visualized with Easyfig 2.2.2 (https://mjsull.github.io/Easyfig/) for Group 1. Genome linear maps are: (1) pBtic235, (2) pBTHD789-2, (3) pAM65-52-3-235K, (4) plasmid 3, (5) pBC244. Functional gene groups are indicated in different colors (see the legend). The gray regions between the genome maps indicate the level of identity from 40 to 100% (see the legend on the right). The numbers of genomes belonging to proven active plasmid prophages are highlighted in yellow and putative active plasmid prophages in green. Identical genomes are marked with blue diamonds. The genomes left inside the brackets are identical and hereafter considered a single genome. The pBtic235 genome was not functionally reannotated in this work and was only used for comparison purposes.

The group includes four sequences with an average genome length of 237,800 bp (Fig. 2, Table S1), three of which, namely plasmid pAM65-52-3-235K^37^, plasmid pBTHD789-2 ]^38^, and plasmid 3^39^, have been described previously by Gillis et al. as sharing more than a 99% nucleotide sequence identity with the pBtic235 molecule from *B. thuringiensis* subsp. *israelensis* GBJ002 (accession number CP051859.1)^40^. The plasmid pBtic235 contains 235,425 bp and was even shown to be an intact prophage as it was able to form turbid plaques on the lawn of a sensitive *B. thuringiensis* strain when induced from the host strain with mitomycin C, as well as to lysogenize the sensitive strain during infection which was proven by PCR with 3 pairs of primers and by Southern blot hybridization^40^.

It was also mentioned by the authors that pBtic235 or its variants had been detected in all the *B. thuringiensis* subsp. *israelensis* genomes sequenced before 2017 but not in other sequenced members of the *B. cereus* group, so these molecules were considered specific to *B. thuringiensis* subsp. *israelensis*. Despite that, the highly related 244,929-bp plasmid pBC244, the fourth member of this group, was reported later in 2017 in *B. cereus* strain BC-AK. It differs from pBtic235 mainly in the small region indicated with the black arrow in Fig. 2 which has no homologues in pBtic235 and contains genes coding for a multi-subunit ABC-type transporter.

Due to the high similarity between pBC244 and the other three plasmids it is quite reasonable to suggest that the former is a pBtic235-related intact prophage although it was deposited in GenBank as a plasmid. All the genomes contain structural gene modules typical of phages with a *Myoviridae*-type tail structure and consisting of genes, coding for tail sheath protein and the putative sheath terminator, three copies of tail tube protein, two tail assembly chaperones, several baseplate components including Mu GpP-like, T4 Gp25-like and P2 GpJ-like proteins and four to six proteins with the putative cell wall adhesion function, one of which resembles bacterial intimin and the others contain either carbohydrate-binding or fibronectin type-III domains.

No tape measure protein (TMP) genes were detected although there is a CDS in all four genomes coding for a protein with the length of 2161 aa and prevailing $\alpha$-helical content according to PSIPRED^41^ prediction, which is believed to function as tape measure protein. All four genomes possess a gene coding for Xer family site-specific tyrosine recombinase highly similar to bacterial recombinase XerS and two genes neighboring each other encoding ParM-like protein and DNA-binding RHH domain-containing protein. Replication-related proteins include two DNA helicases, DNA primase, RuvC-like Holliday junction resolvase, single-stranded (ss) DNA-binding proteins and $\alpha$-subunit of the DNA polymerase III.

### Group 2

**Figure 3:**
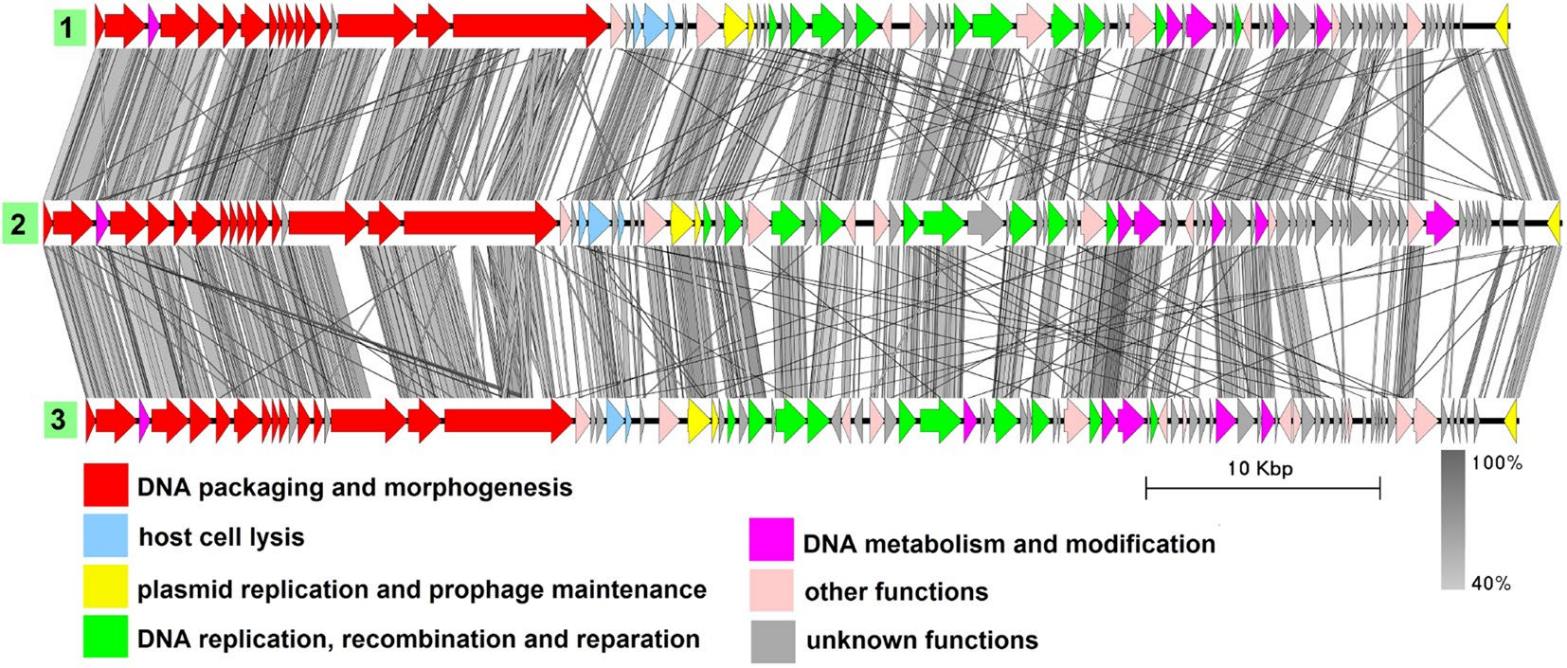
The TBLASTX genome comparison performed and visualized with Easyfig 2.2.2 (https://mjsull.github.io/Easyfig/) for Group 2. Genome linear maps are: (1) pBwiPL1-3, (2) pBWB403, (3) plasmid p2. The functional gene groups are indicated in different colors (see the legend). The gray regions between the genome maps indicate the level of identity from 40 to 100% (see the legend on the right). The numbers of genomes belonging to putative active plasmid prophages are highlighted in green.

This group includes three genomes from three different species: plasmid pBwiPL1-3 from *B. cereus*^42^, plasmid pBWB403 from *B. weihenstephanensis*^43^, and plasmid p2 from *B. thuringiensis*^44^ (Fig. 3, Table S2). The average genome length is 62,269 bp. The plasmids do not show significant similarity to any known viruses in the NCBI nucleotide collection database (taxid:10239) and there have been no tests to show whether these molecules are indeed (or whether they even carry) intact prophages. Despite not being highly identical to each other at the nucleotide level (BLASTN identity values about 70%), the genomes have a similar gene order with minor differences. They possess the structural gene module characteristic of phages with the *Siphoviridae*-type tail structure. Plasmid partitioning proteins are represented by a ParM-like protein and a small RHH domain-containing DNA-binding protein. In each genome, there is a gene coding for a site-specific tyrosine recombinase highly similar to Xer family recombinases, which may be a functional homolog of XerS. Replication-related proteins functionally assigned, include a replication initiator, a helicase loader and the helicase, a DnaG-type primase, a 3$^{\prime }$–5$^{\prime }$ exonuclease, a 5$^{\prime }$–3$^{\prime }$ DNA polymerase and a RuvC-type Holliday junction resolvase.

### Group 3 (B83-like)

**Figure 4:**
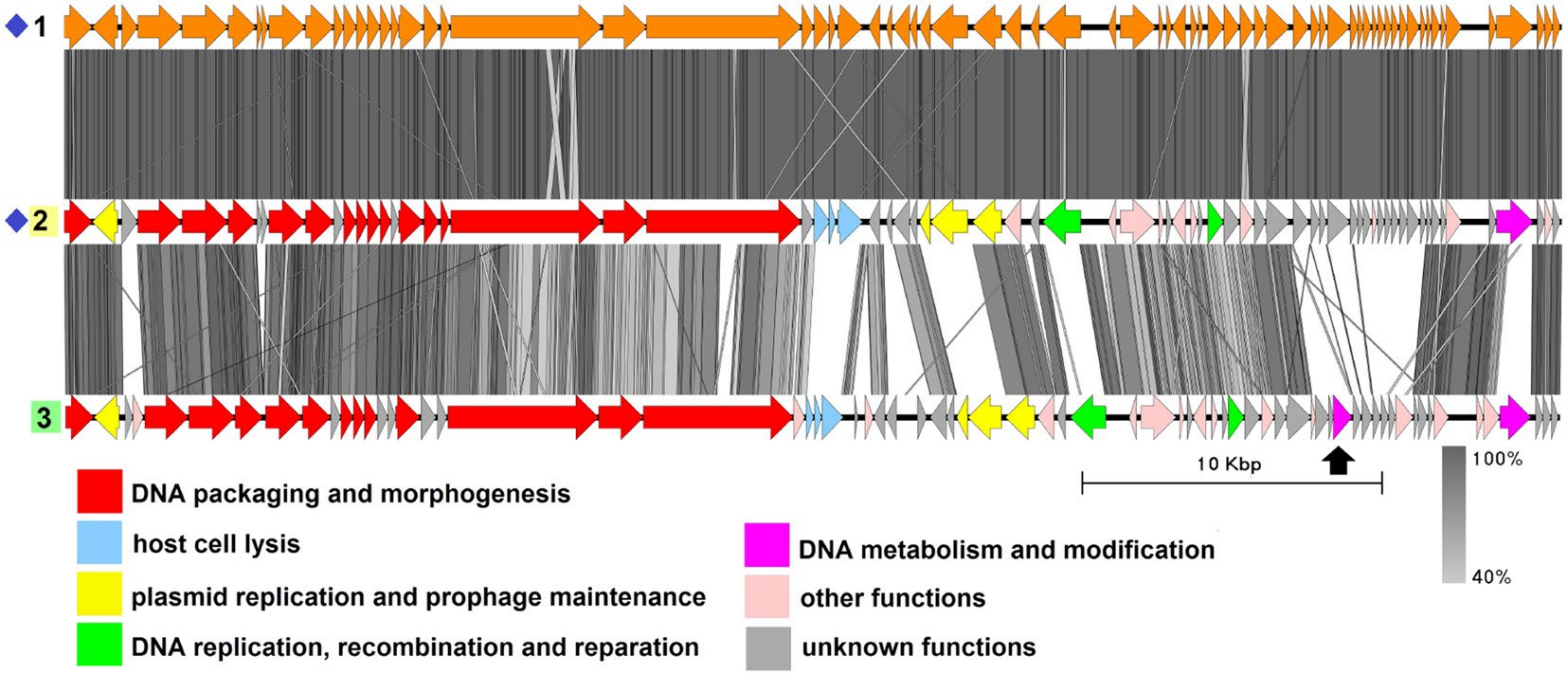
The TBLASTX genome comparison performed and visualized with Easyfig 2.2.2 (https://mjsull.github.io/Easyfig/) for Group 3. Genome linear maps are: (1) vB_BtS_B83, (2) plasmid unnamed2, (3) pBTHD521-2. The functional gene groups are indicated in different colors (see the legend). The gray regions between the genome maps indicate the level of identity from 40 to 100% (see the legend on the right). Identical genomes are marked with blue diamonds. The numbers of genomes belonging to proven active plasmid prophages are highlighted in yellow and putative active plasmid prophages in green. The B83 genome was not functionally reannotated in this work and was only used for comparison purposes.

Group 3 includes two genomes with an average genome length of 49,895 bp: plasmid unnamed2 from the melanin-producing strain *B. thuringiensis* L-7601^42^ and plasmid pBTHD521-2 from *B. thuringiensis* HD521^45^ (Fig. 4, Table S3), with the former being 100% identical to the *Bacillus*-infecting bacteriophage vB_BtS_B83, as described in our previous work^17^.

B83 is a temperate phage with *Siphoviridae*-type tail structure, which possesses two Xer family recombinases and a partitioning system including a ParM-like protein, an RHH domain-containing adapter protein and a putative centromere region, enabling the phage to replicate as a circular plasmid in the prophage stage. Likewise, pBTHD521-2 contains genes coding for Xer recombinases and an actin-like segregation system and is highly similar to the plasmid unnamed2 on the region coding for structural proteins, suggesting it is very likely to be a related temperate phage. There are some minor differences in pBTHD521-2 compared to plasmid unnamed2, mainly with regard to the location and number of small CDSs with unknown function, with the only noticeable exception of the dUTPase gene indicated with the black arrow in Fig. 4 which is absent in plasmid unnamed2. Replication-related proteins are represented by a replication initiator and a RecU-like Holliday junction resolvase.

### Group 4 (phiS58-like)

**Figure 5:**
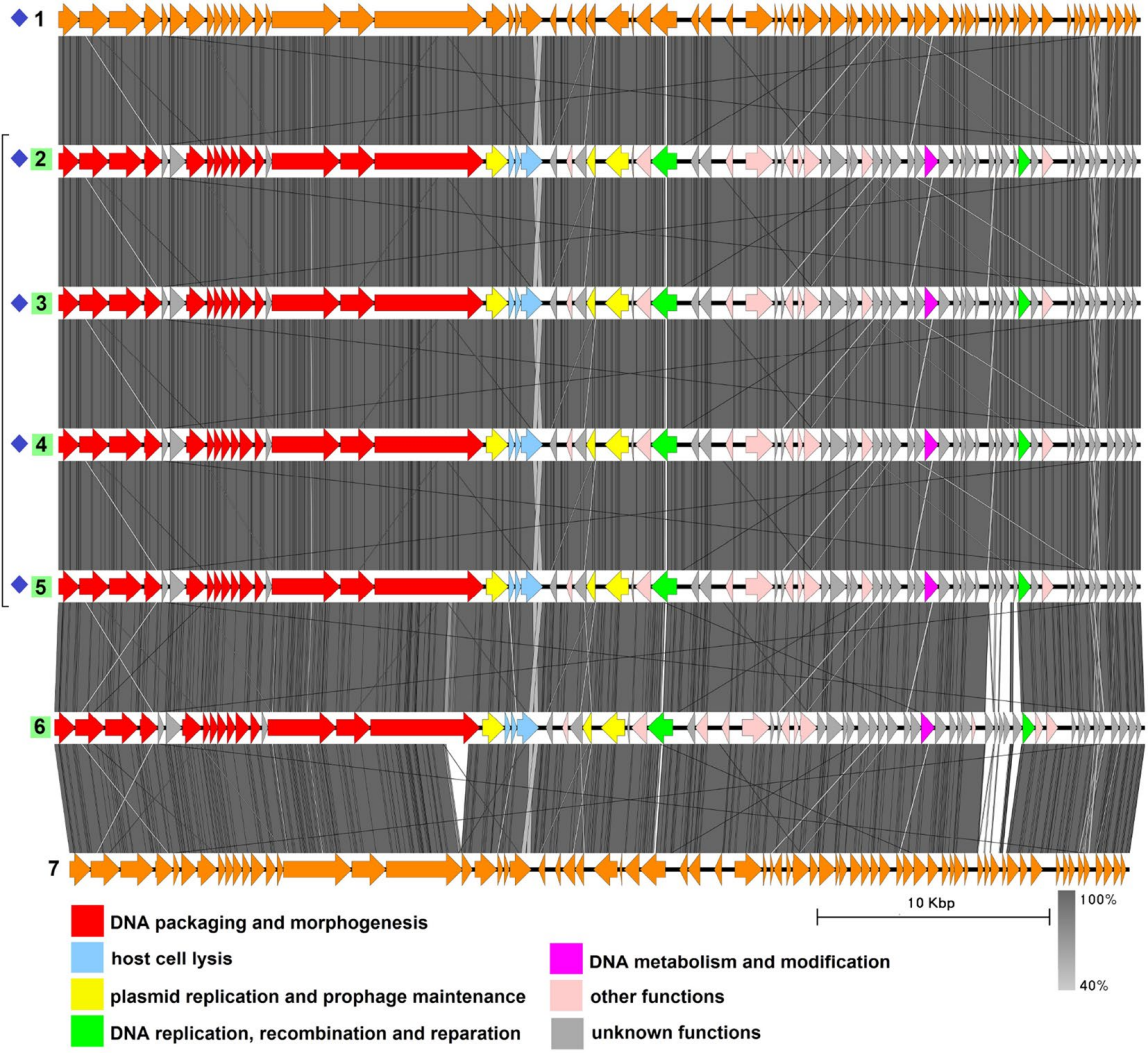
The TBLASTX genome comparison performed and visualized with Easyfig 2.2.2 (https://mjsull.github.io/Easyfig/) for Group 4. The genome linear maps are: (1) phiS58, (2) pYC4, (3) pBMB46, (4) plasmid unnamed7, (5) pYWC2-8-5, (6) pBMB47, (7) Waukesha92. The functional gene groups are indicated in different colors (see the legend). The gray regions between the genome maps indicate the level of identity from 40 to 100% (see the legend on the right). The numbers of genomes belonging to proven active plasmid prophages are highlighted in yellow and putative active plasmid prophages in green. Identical genomes are marked with blue diamonds. The genomes left inside the brackets are identical and hereafter considered a single genome. phiS68 and Waukesha92 genomes were not functionally reannotated in this work and were only used for comparison purposes.

This group includes five genomes with an average length of 46,703 bp, all from the *B. thuringiensis* species (Fig. 5, Table S4). Four of them namely, plasmid pYC4^46^, plasmid pBMB46^47^, plasmid unnamed7^48^ and plasmid pYWC2-8-5^49^ are completely identical to each other and to bacteriophage phiS58, and another, plasmid pBMB47, is slightly different and was reported to be 99% identical to *Bacillus* phage Waukesha92^50^. Structural genes identified are characteristic of phages with *Siphoviridae* morphotype. In each genome there are genes coding for a Xer family recombinase and partitioning proteins: a ParM-like protein and an RHH domain-containing adapter protein. DNA replication, recombination and reparation genes identified include those coding for replication initiator protein and a RecU-like Holliday junction resolvase.

Although Waukesha92 was originally described as belonging to the *Myoviridae* family^51^, we strongly believe that it is a siphovirus as indicated on its GenBank page since its structural gene module is highly similar to those of other members of the group (Fig. 5) and does not encode proteins typical of myoviruses such as the sheath protein, the sheath terminator and specific baseplate proteins, but contains a gene coding for a distal tail protein, which is a highly conserved feature of siphoviruses infecting Gram-positive bacteria^52^.

We could not find a study describing the phiS58 phage, nevertheless, from the information contained in its GenBank file (la_host = “*Bacillus cereus* 6A1”), it appears that the phage was isolated and propagated before sequencing, meaning that it was intact. Considering all of the above, all five genomes of the group very likely belong to temperate bacteriophages of the *B. thuringiensis* species.

### Group 5

**Figure 6:**
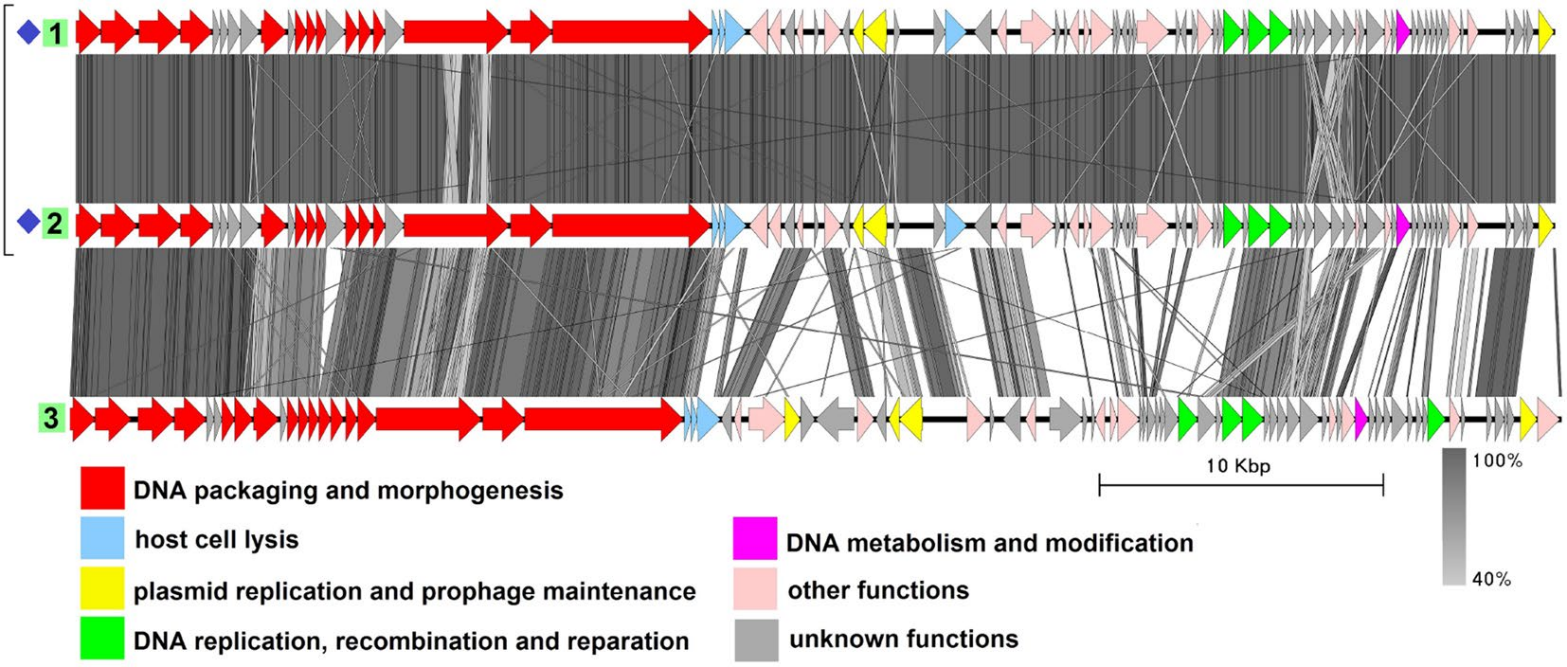
The TBLASTX genome comparison performed and visualized with Easyfig 2.2.2 (https://mjsull.github.io/Easyfig/) for Group 5. The genome linear maps are: (1) pBCN, (2) pBFH_1, (3) pBC52. The functional gene groups are indicated in different colors (see the legend). The gray regions between the genome maps indicate the level of identity from 40 to 100% (see the legend on the right). The numbers of genomes belonging to putative active plasmid prophages are highlighted in green. Identical genomes are marked with blue diamonds. The genomes left inside the brackets are identical and hereafter considered a single genome.

Group 5 includes three genomes, all from the *B. cereus* species: plasmid pBCN^48^, plasmid pBFH_1^39^ and plasmid pBC52 with the former two being identical to one another (Fig. 6, Table S5). The average genome length is 52,342 bp.

DNA packaging and structural genes are highly similar between the genomes. Tail genes identified encode components characteristic of phages with a *Siphoviridae*-type tail structure. Plasmid partitioning proteins are represented by a ParA-Ib-type ATPase and an RHH domain-containing adapter protein. In the pBC52 genome there is a gene coding for a Replix_Relax superfamily protein, which may also be involved in plasmid replication.

Replication-related proteins include ERF-like ssDNA-annealing protein, a replication initiator and a helicase loader. Besides these, pBC52 encodes the RecU-like Holliday junction resolvase.

The pBCN and pBFH_1 genomes encode tyrosine recombinases similar to Xer family recombinases whereas pBC52 has an unrelated 195-aa small serine recombinase which contains an N-terminal serine recombinase domain and a C-terminal small HTH domain suggesting it acts as a resolvase or invertase^8^.

### Group 6

**Figure 7:**
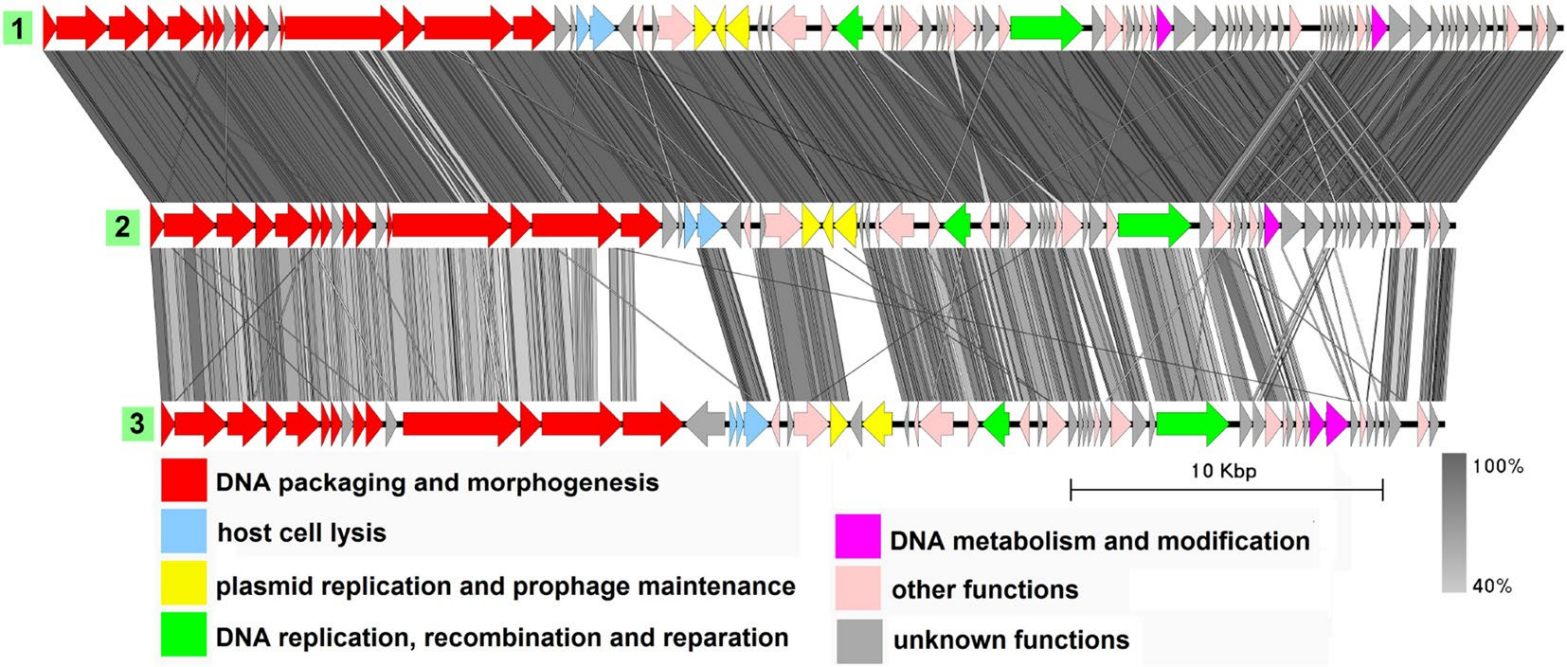
The TBLASTX genome comparison performed and visualized with Easyfig 2.2.2 (https://mjsull.github.io/Easyfig/) for Group 6. The genome linear maps are: (1) plasmid p.6, (2) pBT1850042, (3) pl41. The functional gene groups are indicated in different colors (see the legend). The gray regions between the genome maps indicate the level of identity from 40 to 100% (see the legend on the right above). The numbers of genomes belonging to putative active plasmid prophages are highlighted in green.

This group includes three genomes from *B. thuringiensis* and *B. mycoides*: plasmid p.6, plasmid pBT1850042^53^ and plasmid pl41 (Fig. 7, Table S6) with an average length of 44,010 bp. The first two are highly similar to each other with the exception that there is a 6912-bp repeat in the plasmid p.6 from *B. thuringiensis* QZL38. This may be due to an assembly or sequencing mistake, as we found two more plasmids with even longer repeats, both from the QZL38 strain (described in groups 10 and 11).

Tail genes are characteristic of phages with *Siphoviridae*-type tail structure. Plasmid replication-related proteins of the first two plasmids include a Replix_Relax superfamily protein, a ParA-Ib-type ATPase and an RHH domain-containing protein. Instead of the latter two, the pl41 plasmid possesses a ParM-like protein and a small protein with weak homology to different RHH domain-containing proteins. Replication-related proteins include a replication initiator and a primase-polymerase (Primpol) domain-containing protein. All three genomes contain dUTPase genes and in the plasmid pl41 there is also a thymidylate synthase gene located immediately downstream from the dUTPase.

### Group 7

**Figure 8:**
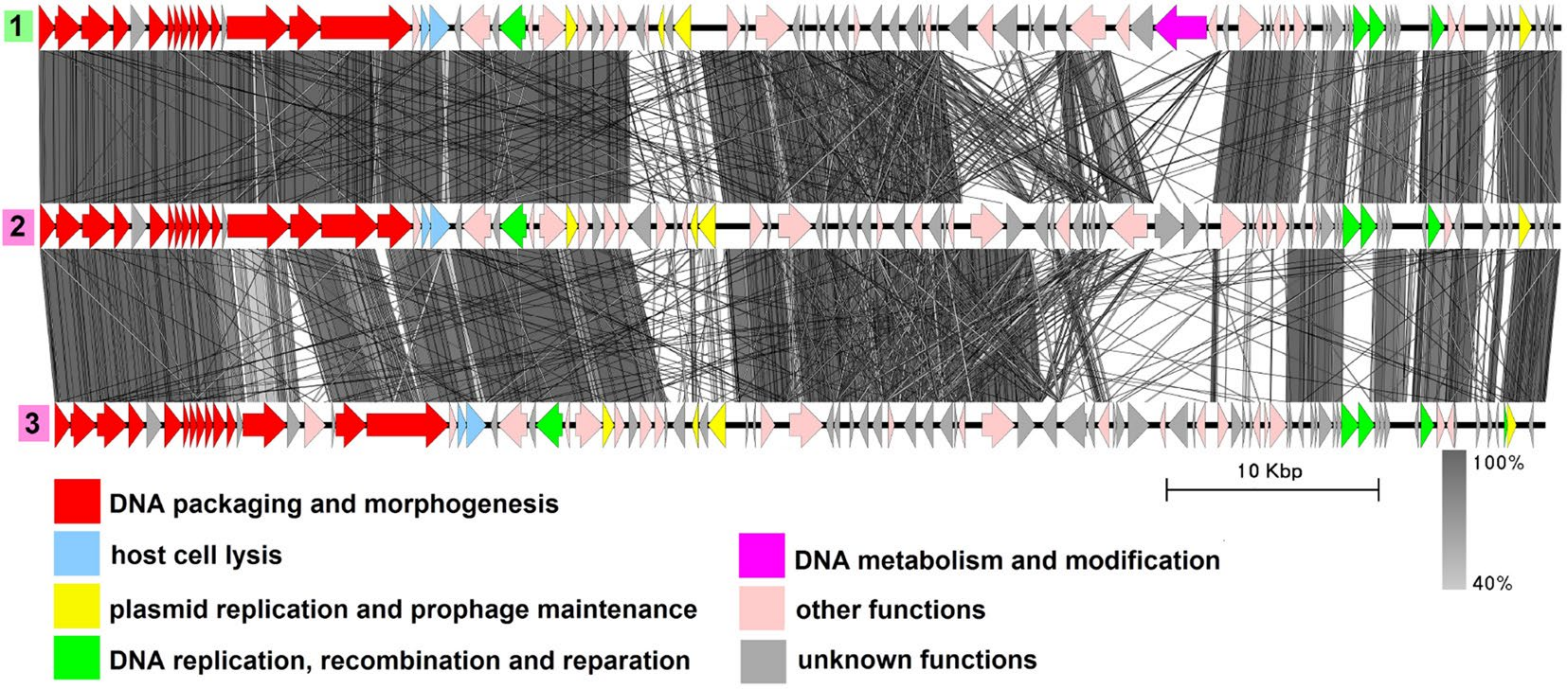
The TBLASTX genome comparison performed and visualized with Easyfig 2.2.2 (https://mjsull.github.io/Easyfig/) for Group 7. The genome linear maps are: (1) pBCK802, (2) pBTHD521-3, 3) pBCA2. The functional gene groups are indicated in different colors (see the legend). The gray regions between the genome maps indicate the level of identity from 40 to 100% (see the legend on the right). The numbers of genomes belonging to putative active plasmid prophages are highlighted in green and the putative degenerated plasmid prophages in pink.

This group includes three plasmids from *B. cereus* and *B. thuringiensis* species: plasmid pBCK802, plasmid pBTHD521-3^45^ and plasmid pBCA2^54^ (Fig. 8, Table S7) with an average length of 71,333 bp. Tail components are typical of phages with *Siphoviridae* morphotype. The baseplate hub protein-coding gene in the pBTHD521-3 genome, and the TMP gene in the pBCA2 genome are each split into two CDSs, suggesting these genomes are probably not able to encode functional phage particles unless the stop codons appeared due to frameshifting sequencing errors. All the plasmids encode a Replix_Relax superfamily protein, a ParA-Ib-type ATPase and an RHH domain-containing putative adapter protein. Replication proteins include a replication initiator, a helicase loader and a RecU-like Holliday junction resolvase. Also, all the genomes encode 192-aa small site-specific serine recombinase (Resolvase and Invertase subfamily). In the pBCK802 genome there is also a gene coding for a protein with an N-terminal N-6 adenine-specific DNA methylase domain and a C-terminal domain which is similar to type II restriction endonucleases BsuBI and PstI.

### Group 8

**Figure 9:**
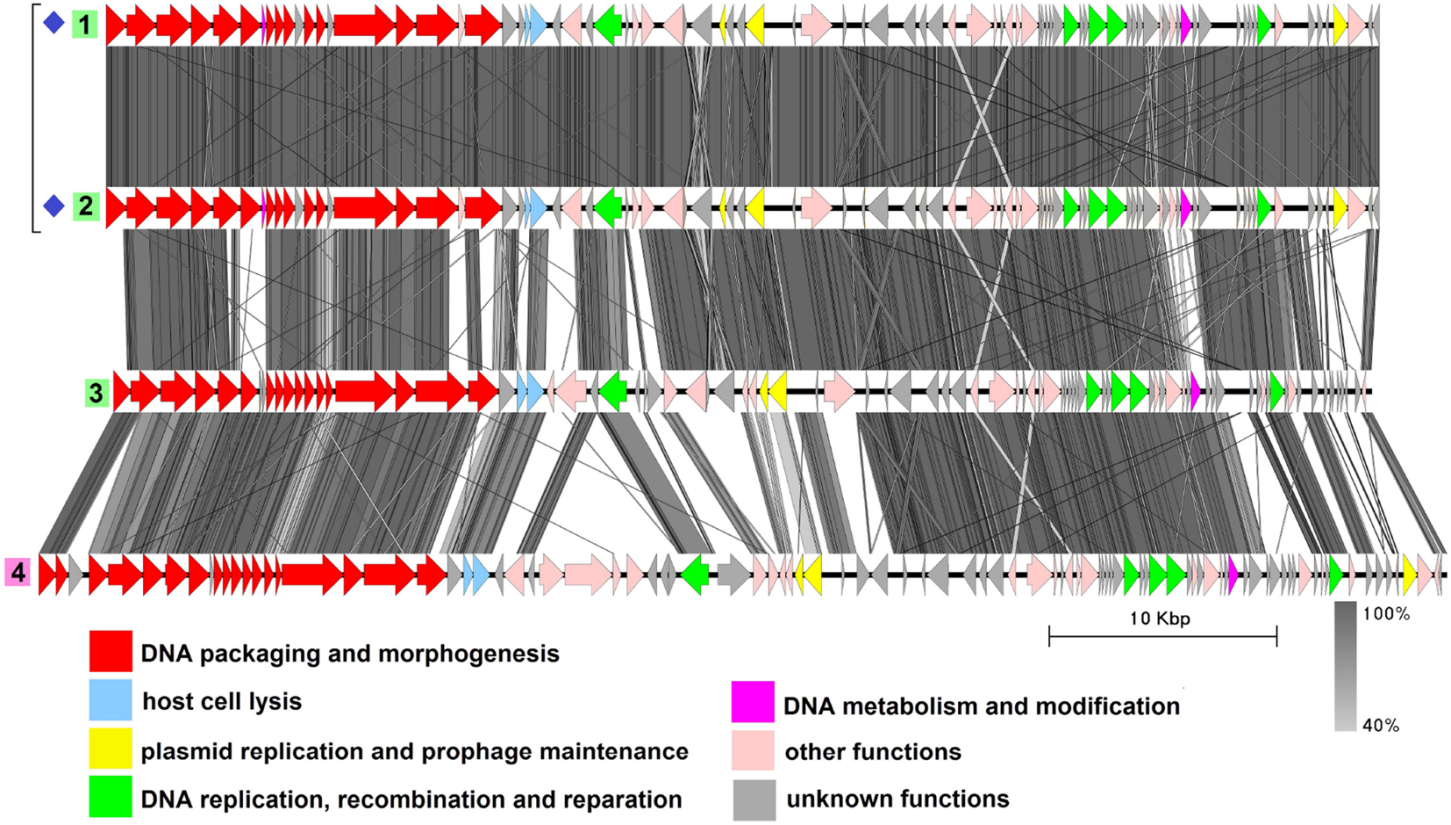
The TBLASTX genome comparison performed and visualized with Easyfig 2.2.2 (https://mjsull.github.io/Easyfig/) for Group 8. The genome linear maps are: (1) pALH1, (2) pBFQ, (3) pF837_55, (4) pBFI_4. The functional gene groups are indicated in different colors (see the legend). The gray regions between the genome maps indicate the level of identity from 40 to 100% (see the legend on the right). The numbers of genomes belonging to putative active plasmid prophages are highlighted in green and putative degenerated plasmid prophages in pink. Identical genomes are marked with blue diamonds. The genomes left inside the brackets are identical and hereafter considered a single genome.

Group 8 includes plasmids from the *B. thuringiensis* and the *B. cereus* species: plasmid pALH1 which was described as a ‘circular phage’ in the original study^55^ but has not been experimentally proven, plasmid pF837_55, reported as a putative nonintegrated prophage because of its multiple phage-related genes^56^, plasmid pBFQ^39^ and plasmid pBFI_4 (Fig. 9, Table S8)^39^. The average length of the plasmids is 57,261 bp.

The tail components identified are typical of phages with *Siphoviridae* morphotype. Plasmid replication-related proteins are: a ParA-Ib-type ATPase and an RHH domain-containing DNA-binding protein. Replication-related proteins shared by all four genomes are: the UmuC-like subunit of translesion synthesis DNA polymerase, the ERF family ssDNA-annealing protein, a replication initiator, the putative DnaC-type helicase loader and a RecU-like Holliday junction resolvase. Except for the plasmid pF837_55, all the genomes also possess a small site-specific serine recombinase (Resolvase and Invertase subfamily). In the pBFI_4 genome, the large terminase subunit gene is truncated compared to that of the rest of the three genomes, suggesting that pBFI_4 is probably a non-functional prophage remnant.

### Group 9

**Figure 10:**
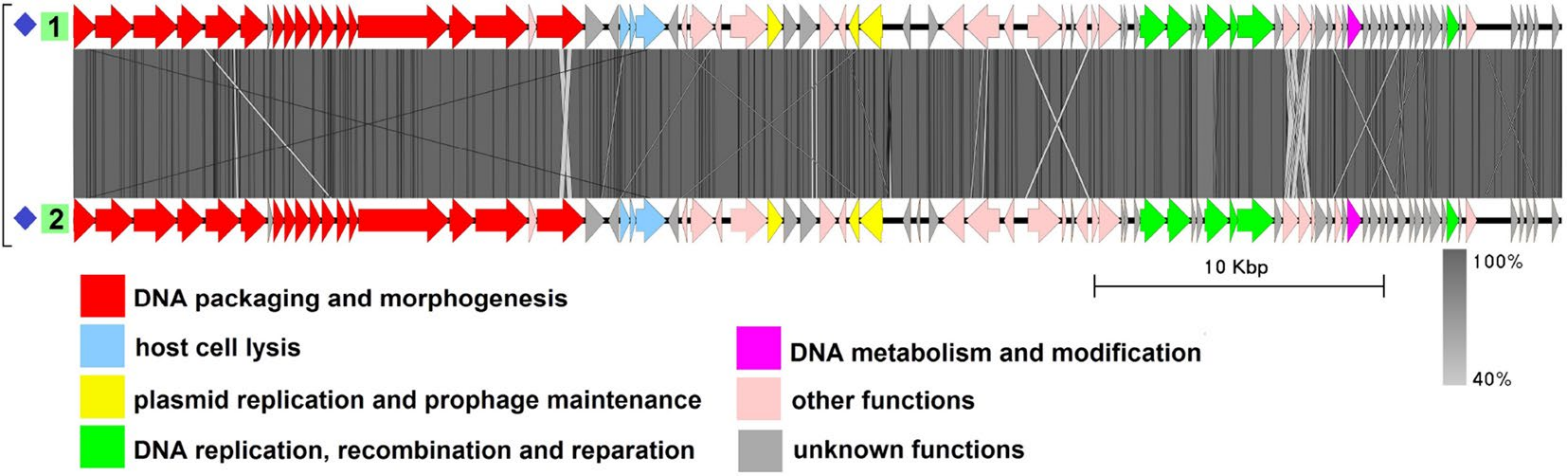
The TBLASTX genome comparison performed and visualized with Easyfig 2.2.2 (https://mjsull.github.io/Easyfig/) for Group 9. The genome linear maps are: (1) pBFC_2, (2) pBFR_3. The functional gene groups are indicated in different colors (see the legend). The gray regions between the genome maps indicate the level of identity from 40 to 100% (see the legend on the right). The numbers of genomes belonging to putative active plasmid prophages are highlighted in green. Identical genomes are marked with blue diamonds. The genomes left inside the brackets are identical and hereafter considered a single genome.

This group includes two plasmids almost identical to each other, both from the *B. cereus* species: plasmid pBFC_2^39^ and plasmid pBFR_3 (Fig. 10, Table S9). The average genome length is 51,522 bp.

Tail proteins are typical of phages with *Siphoviridae* morphotype. Proteins related to plasmid replication include a Replix_Relax superfamily protein, a ParA-Ib-type ATPase and an RHH domain-containing protein. Other replication and recombination-related proteins include a YqaJ domain-containing exonuclease and a RecT-like ssDNA-annealing protein, parts of a two-component recombination system functionally similar to SPP1 Gp34.1 and Gp35, as well as a replication initiator, a G39P-like helicase loader/inhibitor, a $\hbox {DnaB}_{Eco}$-type replicative DNA helicase and a RusA-like Holliday junction resolvase.

### Group 10

**Figure 11:**
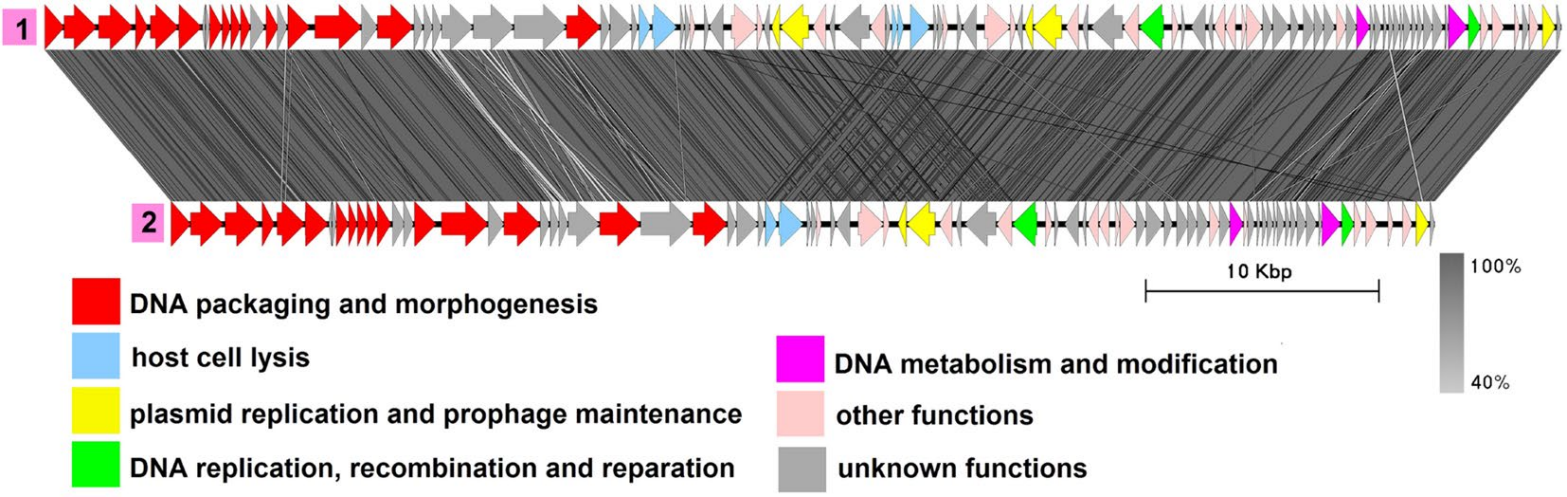
The TBLASTX genome comparison performed and visualized with Easyfig 2.2.2 (https://mjsull.github.io/Easyfig/) for Group 10. The genome linear maps are: (1) plasmid p.3, (2) pBT1850054. The functional gene groups are indicated in different colors (see the legend). The gray regions between the genome maps indicate the level of identity from 40 to 100% (see the legend on the right). The numbers of genomes belonging to putative degenerated plasmid prophages are highlighted in pink.

Group 10 includes plasmid p.3 and plasmid pBT1850054^53^ (Fig. 11, Table S10) from *B. thuringiensis*. The plasmids are identical to each other with the exception that there is a 10,852 bp-repeat in plasmid p.3 obtained from *B. thuringiensis* QZL38. Two more repeat-containing plasmids (described in Group 6 and Group 11) were also obtained from the QZL38 strain, suggesting these repeats are likely to have resulted from sequencing or assembly mistakes.

The average genome length is 59,631 bp. Tail components are characteristic of phages with *Siphoviridae* morphotype. Besides a putative tail terminator protein, a tail tube protein, a baseplate hub protein and a putative cell adhesion protein containing a cellulose-binding domain, the tail components include four proteins with unknown functions, similar to those annotated as ‘structural proteins’ from *Brevibacillus* phage Jenst, a member of the *Siphoviridae* family. The putative TMP gene is split into two CDSs coding for products with lengths of 313 and 669 aa residues, which may not be functional, though still show significant similarity to proteins annotated as TMP from various phages. Both genomes possess genes coding for a ParM-like protein and a small RHH domain-containing protein, and also a gene coding for a site-specific tyrosine recombinase. Replication-related proteins include a replication initiator and a RecU-like Holliday junction resolvase. DNA modification proteins are represented by a dUTPase and a site-specific DNA-adenine methylase.

### Group 11

**Figure 12:**
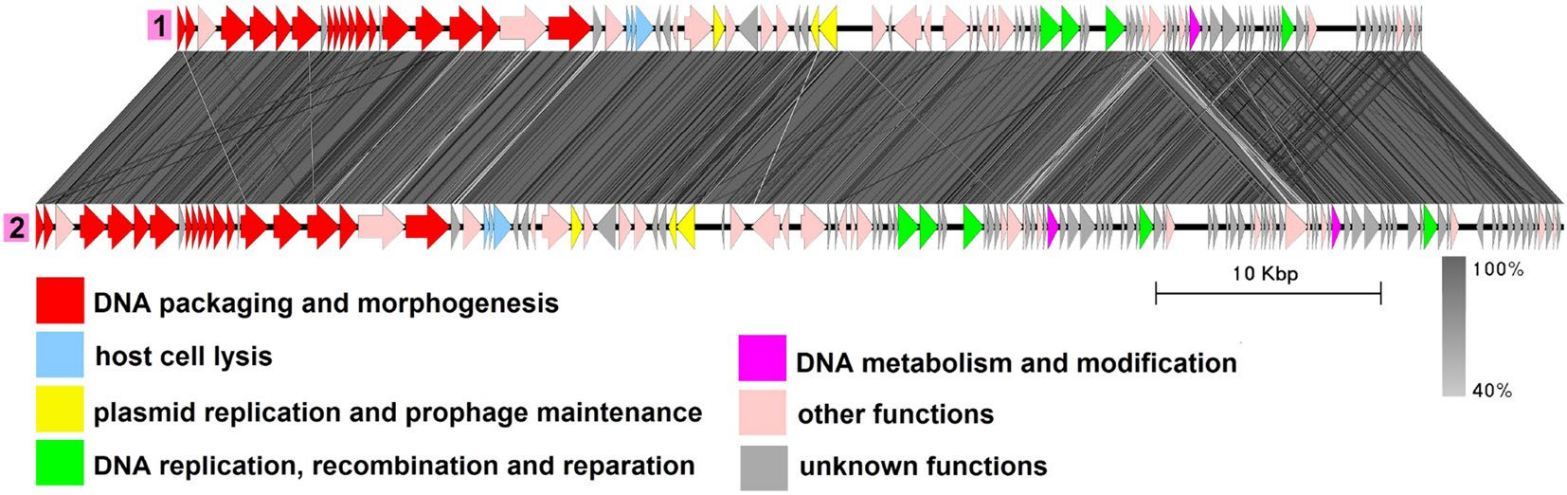
The TBLASTX genome comparison performed and visualized with Easyfig 2.2.2 (https://mjsull.github.io/Easyfig/) for Group 11. The genome linear maps are: (1) pBT1850055, (2) plasmid p.4. The functional gene groups are indicated in different colors (see the legend). The gray regions between the genome maps indicate the level of identity from 40 to 100% (see the legend on the right). The numbers of genomes belonging to putative degenerated plasmid prophages are highlighted in pink.

Group 11 includes plasmid pBT1850055^53^ and plasmid p.4 (Fig. 12, Table S11) from *B. thuringiensis*. The second plasmid was obtained from the above-mentioned QZL38 strain and, except for the 12,650 bp-repeat, is identical to plasmid pBT1850055.

The average genome length is 61,697 bp. Structural genes are typical of *Siphoviridae* morphotype. In both genomes there are three CDSs coding for products (415, 429 and 480 aa) similar to TMPs from different *Bacillus*-infecting phages, but considering that they are significantly shorter than typical TMP from phages with medium size genomes, they are probably just remnants of once functional genes unless the splitting is caused by frameshifting sequencing errors. Plasmid replication-related proteins include a Replix_Relax superfamily protein, a ParA-Ib-type ATPase and an RHH domain-containing protein. Replication-related proteins identified include the YqaJ domain-containing exonuclease, the RecT-like ssDNA-annealing protein, the helicase loader and the RecU-like Holliday junction resolvase.

### Singletons

Twenty-five plasmids were not assigned to any group. Among these, 12 were classified as putative plasmid prophages, 10 – putative degenerated plasmid prophages, one – a proven plasmid prophage, one – a proven plasmid-integrated prophage and another one – a putative virulent phage (Fig. S1, Table S12).

Plasmid pBWB404 from *B. weihenstephanensis* KBAB4 was assumed to be an extrachromosomal prophage^43^ but it is probably a degenerated plasmid prophage with its TMP gene split into two CDSs coding for 488-aa and 787-aa products.

Plasmid pBMB165 from *B. thuringiensis* serovar *tenebrionis* YBT-1765 was shown to harbor an inducible prophage, BMBTP3^57^. The prophage part of the plasmid has genes coding for Xer family recombinase, a ParM-like protein and an RHH-domain-containing putative adapter protein, while in the rest of the plasmid there are genes for another Xer family recombinase, and another partitioning system including the ParA-Ib-type ATPase and the RHH-domain-containing protein.

Plasmid CP013273.1 from *B. thuringiensis* CTC was claimed to be a linear prophage^58^ although this has not been experimentally proven. The plasmid is significantly related to the *L. lactis* phages P2, jj50 and sk1 (Table S12), as well as to other members of the genus *Skunavirus*, which are known to be virulent phages. Neither plasmid partitioning-related genes nor recombinases were identified in the CP013273.1 genome, suggesting it is highly likely to be a virulent phage as well. Bacteriophage sk1 and some other members of *Skunavirus* have been shown to use the *cos* DNA packaging strategy^59,60^, and since the large terminase amino-acid sequences of sk1 and CP013273.1 are almost identical (BLASTP coverage 100%, identity 98.33%), and it is known that phages with highly similar packaging machineries usually follow the same packaging strategy^61^, CP013273.1 may also be a *cos* phage. If this is true, it may explain the observed linearity of the CP013273.1 contig, which may have been obtained not from a linear plasmid, but from the phage virion DNA that contaminated the sequencing library. This is supported by the fact that cases of *cos* phage sequences resulting in linear contigs with their ends corresponding to the physical termini of phage chromosomes is not unusual^21^, which is in stark contrast to headful phages and those with terminal repeats for which a complete assembly usually results in one circular contig^22^.

Plasmid pNC1 (AP007211.1) from *B. cereus* strain NC7401 was first reported in 2012^62^ and later was proven to be an inducible extrachromosomal prophage, PfNC7401^63^.

## Discussion

The issue of finding extrachromosomal prophages in *Bacillus* was considered in 2015 as a part of extensive work by Simon Roux and others^64^ where microbial genomes were searched for viral sequences using VirSorter^65^, which resulted in the identification of 1756 high-confidence viral sequences represented by complete (circular) and/or large (> 30 kb) extrachromosomal genome fragments, which the authors concluded could belong to: chronic phages, lytic viruses in a ‘carrier’ state or extrachromosomal prophages^64^. Among the 1,756 viral sequences extracted from various bacterial and archaeal genomes, only 108 were tagged in the NCBI database as plasmids. For the *B. cereus sensu lato* group, 14 such ‘plasmids’ were identified by the authors^64^, of which six (CP003188.1, CP003689.1, CP000229.1, CP000486.1, CP005936.1, CP000906.1) have been marked in this study as putative plasmid prophages; one (AP007211.1) is a proven plasmid prophage, two (CP003757.1 and CP000907.1) we identified as putative degenerated plasmid prophages, one (AE016878.2) was excluded from the data set as it is closely related to the known *Bacillus*-infecting tectiviruses AP50 and Bam35c and therefore does not belong to the target *Caudovirales* group, one (CP003767.1) was not initially included in the initial data set as it is slightly shorter (14,935 bp) than the selected lowest limit of 15 kbp, and three (CP001911.1, CP004130.1 and CP001188.1) were discarded at the hmmsearch stage as they did not have matches for terminase and/or major capsid protein profiles.

For the latter three genomes, nucleotide fasta files were downloaded and reannotated to verify the solid reasoning for the exclusion of the genomes. We have proven that the three genomes lack identifiable small and large terminase subunits as well as several known capsid components, although they do possess tail genes and therefore appear to be *Myoviridae* (CP001188.1) and *Siphoviridae* (CP001911.1, CP004130.1) degenerated prophage remnants.

According to the data we have obtained our results are almost completely consistent with the results of previous research. As the number of finished sequencing projects increases and virus-identifying instruments become more powerful, new large-scale search projects will become possible in order to update and expand our knowledge of the actual number of phages hidden in bacterial WGS data and metagenomes. Although large-scale projects appear to be extremely effective for the detection of viral signals in massive mixed genomic datasets, small-scale projects targeted at particular groups of phages are also essential as they facilitate more detailed analyzes and allow us to physically study and review candidate genomes and select those that are highly likely to represent actual phages and therefore may prove to be worth further investigation including but not limited to experimental assays.

Although there is an abundance of computational tools designed for prophage detection in bacterial genomes^66^, experimental approaches based on stress induction remain the optimum method for discovering whether a bacterial strain harbors functional prophages, given the fact that the predictive ability of the available computational tools remains insufficient when identifying exact prophage location and uncovering signs of prophage activity^67^.

Stress induction is typically achieved by exposing bacterial culture to stress agents such as mitomycin C, this allows for phage particles to be purified from the obtained lysate, propagated into new cultures and their genome sequence determined partially or completely and then compared to that of previously sequenced host genomes. These approaches therefore facilitate the identification of prophage location, making it possible for plasmid prophages to be distinguished from those integrated in the host chromosome or in a host plasmid^68^. Experimental confirmation may be achieved through Southern blotting analysis of bacterial DNA with probes obtained from the phage DNA, as has been performed by Sakaguchi, Yoshihiko et al.^10^. This is probably the most precise method for enabling the determination of prophage location in a lysogenized host.

A number of studies have been published on circular plasmid prophages that do not include whole-genome sequencing of bacterial hosts or Southern blotting-based analyzes and which use different approaches to proving the existence of plasmid prophages including restriction-based analyses of bacterial plasmid DNA^14,17^. For phages with circular plasmid prophage forms, almost all of the restriction fragments obtained for the phage chromosomal DNA (from capsids) can be found in the restriction patterns of the host plasmid DNA, with minor differences depending on the mode of DNA packaging (*cos*, headful or DTR)^61^.

Other evidence may be obtained by sequencing DNA from phage particles and comparing it with the contigs resulting from total plasmid DNA sequencing. If the plasmid assembly contains a contig identical to the phage genome with read coverage comparable to that of other plasmid contigs, this indicates that the phage is indeed maintained in the host cytoplasm as a circular plasmid^69^. These two approaches are cheap and simple but have an important limitation: although they can prove the existence of circular plasmid prophages they can not exclude the possibility that the prophages can also integrate into the host DNA.

In contrast with the issue of proving the existence of plasmid prophages, distinguishing between truly circular molecules, whether phage-related or not, and artificially circularized contigs obtained from linear phage chromosomes in WGS bacterial projects, can in some cases be achieved computationally by finding local read coverage deviations, constituting evidence against DNA circularity. In metagenomic projects, due to the increasing amount of data, this approach is less realistic, especially with regard to phages with the headful mechanism of DNA packaging, which when completely sequenced, result in circular contigs with minor coverage deviations. This makes metagenomics-derived phage sequences less reliable than those from viral metagenomics projects (which include filtration stages to remove all objects larger in diameter than most viruses) resulting in sequencing data that only includes reads obtained from encapsidated DNA but not prophages. This difference is vitally important when aiming to uncover phage diversity and obtain reliable sequences belonging to currently existing functional phages, e.g., in order to improve and expand phage taxonomy.

In conclusion, in this study: of the plasmids from the *B. cereus sensu lato* species out of the 474 initially selected based on their size (15–500 kbp) and criteria concerning assembly completeness, only 28 (5.9%) were found to be potential active plasmid prophages. We also identified three genomes belonging to proven plasmid prophages, including one plasmid that was experimentally verified to be an inducible plasmid prophage (AP007211.1), and two genomes identical to known bacteriophages capable of forming plasmid prophages (CP020004.1 identical to bacteriophage B83, and CP003765.1, CP013278.1 and CP009347.1 identical to plasmid pBtic235 and to each other and therefore considered a single genome). Also, 17 genomes were classified as putative degenerated plasmid prophages, one a proven plasmid-integrated prophage and another one a putative virulent phage.

Twenty-eight genomes classified as putative plasmid prophages very likely belong to plasmid prophages though some of them may also be prophages integrated into small plasmids, which are difficult to distinguish from *bona fide* plasmid prophages. Whether they are still inducible and able to produce functional phage particles is another unanswered question that needs to be addressed experimentally. Out of these final 28, 23 are so distinct from already known phages and from each other, that had they been described as viruses, they may have already been proven to be representatives of 23 new genera.

## Supplementary Information

Supplementary Information.
