# Supplementary material for: Putative plasmid prophages of Bacillus cereus sensu lato may hold the key to undiscovered phage diversity
Source: Sci Rep. 2021 Apr 7;11:7611. doi: 10.1038/s41598-021-87111-3 (PMC8026635; doi:10.1038/s41598-021-87111-3)
Supplement: Supplementary file 1 — Supplementary Information. [file 41598_2021_87111_MOESM1_ESM.pdf]

Supplementary Information

Putative plasmid prophages of *Bacillus cereus sensu lato* may hold the key to undiscovered phage diversity

Emma G. Piligrimova[1]*, Olesya A. Kazantseva[1], Andrey N. Kazantsev[2], Nikita A. Nikulin[1], Anna V. Skorynina[1], Olga N. Koposova[1], and Andrey M. Shadrin[1]*

[1]Laboratory of Bacteriophage Biology, G.K. Skryabin Institute of Biochemistry and Physiology of Microorganisms,Pushchino Scientific Center for Biological Research of the Russian Academy of Sciences, Federal Research Center, 142290, Pushchino, Russia

[2]P. N. Lebedev Physical Institute of the Russian Academy of Sciences, Pushchino Radio Astronomy Observatory, Pushchino 142290, Russia

Name	Accession number	Genome size, bp	GC-content, %	Number of protein-coding genes	Number of tRNAs and preudo-tRNAs	Number of proteins shared with the genome by all of the previous genomes of the table	Proteins shared with the genome by all of the previous genomes of the table, % (in terms of the average number of protein-coding genes in the genomes)	Type
B. thuringiensis HD-789 plasmid pBTHD789-2	CP003765.1	235,425	36.6	260	17	-	-	proven plasmid prophage
B. thuringiensis serovar *israelensis* AM65-52 plasmid pAM65-52-3-235K	CP013278.1	235,424	36.6	260	17	-	-	proven plasmid prophage
B. thuringiensis HD1002 plasmid 3	CP009347.1	235,425	36.6	260	17	-	-	proven plasmid prophage
B. cereus BC-AK plasmid pBC244	CP020938.1	244,929	36.5	280	17	227	84.1	putative plasmid prophage

Table S1. General characteristics of Group 1 genomes.

Name	Accession number	Genome size, bp	GC-content, %	Number of protein-coding genes	Number of tRNAs and preudo-tRNAs	Number of proteins shared with the genome by all of the previous genomes of the table	Proteins shared with the genome by all of the previous genomes of the table, % (in terms of the average number of protein-coding genes in the genomes)	Type
B. cereus PL1 plasmid pBwiPL1-3	AP022646.1	60,534	43.7	83	0	-	-	putative plasmid prophage
B. weihenstephanensis KBAB4 plasmid pBWB403	CP000906.1	64,977	43.4	89	0	50	58.1	putative plasmid prophage
B. thuringiensis HM-311 plasmid p2	CP040784.1	61,297	44.2	89	0	45	51.7	putative plasmid prophage

Table S2. General characteristics of Group 2 genomes.

Name	Accession number	Genome size, bp	GC-content, %	Number of protein-coding genes	Number of tRNAs and preudo-tRNAs	Number of proteins shared with the genome by all of the previous genomes of the table	Proteins shared with the genome by all of the previous genomes of the table, % (in terms of the average number of protein-coding genes in the genomes)	Type
B. thuringiensis L-7601 plasmid unnamed2	CP020004.1	49,952	35.8	71	0	-	-	proven plasmid prophage
B. thuringiensis serovar *indiana* HD521 plasmid pBTHD521-2	CP010108.1	49,838	35.9	70	0	44	62.4	putative plasmid prophage

Table S3. General characteristics of Group 3 genomes.

Name	Accession number	Genome size, bp	GC-content, %	Number of protein-coding genes	Number of tRNAs and preudo-tRNAs	Number of proteins shared with the genome by all of the previous genomes of the table	Proteins shared with the genome by all of the previous genomes of the table, % (in terms of the average number of protein-coding genes in the genomes)	Type
B. thuringiensis YC-10 plasmid pYC4	CP011352.1	46,634	35.4	70	0	-	-	putative plasmid prophage
B. thuringiensis serovar *kurstaki* HD-1 plasmid pBMB46	CP004871.1	46,634	35.4	70	0	-	-	putative plasmid prophage
B. thuringiensis serovar *kurstaki* HD 1 plasmid unnamed7	CP010006.1	46,634	35.4	70	0	-	-	putative plasmid prophage
B. thuringiensis YWC2-8 *plasmid* pYWC2-8-5	CP013060.1	46,634	35.4	70	0	-	-	putative plasmid prophage
B. thuringiensis serovar *galleriae* HD-29 plasmid pBMB47	CP010095.1	46,979	35.5	71	0	66	93.6	putative plasmid prophage

Table S4. General characteristics of Group 4 genomes.

Name	Accession number	Genome size, bp	GC-content, %	Number of protein-coding genes	Number of tRNAs and preudo-tRNAs	Number of proteins shared with the genome by all of the previous genomes of the table	Proteins shared with the genome by all of the previous genomes of the table, % (in terms of the average number of protein-coding genes in the genomes)	Type
B. cereus 03BB87 plasmid pBCN	CP009939.1	52,166	36.5	86	0	-	-	putative plasmid prophag e
B. cereus G9241 plasmid pBFH_1	CP009589.1	52,166	36.5	86	0	-	-	putative plasmid prophag e
B. cereus BC-AK plasmid pBC52	CP020939.1	52,693	36.6	80	0	49	59.0	putative plasmid prophag e

Table S5. General characteristics of Group 5 genomes.

Name	Accession number	Genome size, bp	GC-content, %	Number of protein-coding genes	Number of tRNAs and preudo-tRNAs	Number of proteins shared with the genome by all of the previous genomes of the table	Proteins shared with the genome by all of the previous genomes of the table, % (in terms of the average number of protein-coding genes in the genomes)	Type
B. thuringiensis QZL38 plasmid p.6	CP032613.1	48,849	35.2	86	0	-	-	putative plasmid prophage
B. thuringiensis Bt185 plasmid pBT1850042	CP014287.1	41,937	35.3	69	0	-	-	putative plasmid prophage
B. mycoides BPN401 plasmid pl41	CP031074.1	41,246	34.8	60	0	40	62.0	putative plasmid prophage

Table S6. General characteristics of Group 6 genomes.

Name	Accession number	Genome size, bp	GC-content, %	Number of protein-coding genes	Number of tRNAs and preudo-tRNAs	Number of proteins shared with the genome by all of the previous genomes of the table	Proteins shared with the genome by all of the previous genomes of the table, % (in terms of the average number of protein-coding genes in the genomes))	Type
B. cereus K8 plasmid pBCK802	CP016597.1	71,854	34.4	91	0	91	-	putative plasmid prophage
B. thuringiensis serovar *indiana* s HD521 plasmid pBTHD521-3	CP010109.1	71,771	34.4	95	0	71	76.3	putative degenerated plasmid prophage
B. cereus A1 plasmid pBCA2	CP015728.1	70,374	34.5	97	0	56	59.4	putative degenerated plasmid prophage

Table S7. General characteristics of Group 7 genomes.

Name	Accession number	Genome size, bp	GC-content, %	Number of protein-coding genes	Number of tRNAs and preudo-tRNAs	Number of proteins shared with the genome by all of the previous genomes of the table	Proteins shared with the genome by all of the previous genomes of the table, % (in terms of the average number of protein-coding genes in the genomes)	Type
B. thuringiensis Al Hakam, plasmid pALH1	CP000486.1	55,939	36.2	86	0	-	-	putative plasmid prophage
B. thuringiensis HD571 plasmid pBFQ	CP009599.1	55,939	36.2	86	0	-	-	putative plasmid prophage
B. cereus F837/76 plasmid pF837_55	CP003188.1	55,304	36.2	90	0	66	75.0	putative plasmid prophage
B. cereus 03BB108 plasmid pBFI_4	CP009638.1	61,862	35.5	95	0	55	61.0	putative degenerated plasmid prophage

Table S8. General characteristics of Group 8 genomes.

Name	Accession number	Genome size, bp	GC-content, %	Number of protein-coding genes	Number of tRNAs and preudo-tRNAs	Number of proteins shared with the genome by all of the previous genomes of the table	Proteins shared with the genome by all of the previous genomes of the table, % (in terms of the average number of protein-coding genes in the genomes)	Type
B. cereus 3a plasmid pBFC_2	CP009594.1	51,531	36.6	84	0	-	-	putative plasmid prophage
B. cereus S2-8 plasmid pBFR_3	CP009603.1	51,512	36.6	84	0	84	100.0	putative plasmid prophage

Table S9. General characteristics of Group 9 genomes.

Name	Accession number	Genome size, bp	GC-content, %	Number of protein-coding genes	Number of tRNAs and preudo-tRNAs	Number of proteins shared with the genome by all of the previous genomes of the table	Proteins shared with the genome by all of the previous genomes of the table, % (in terms of the average number of protein-coding genes in the genomes)	Type
B. thuringiensis QZL38 plasmid p.3	CP032610.1	65,057	34.4	102	0	-	-	putative degenerated plasmid prophage
B. thuringiensis Bt185 plasmid pBT1850054	CP014286.1	54,205	34.7	82	0	-	-	putative degenerated plasmid prophage

Table S10. General characteristics of Group 10 genomes.

Name	Accession number	Genome size, bp	GC-content, %	Number of protein-coding genes	Number of tRNAs and preudo-tRNAs	Number of proteins shared with the genome by all of the previous genomes of the table	Proteins shared with the genome by all of the previous genomes of the table, % (in terms of the average number of protein-coding genes in the genomes)	type
B. thuringiensis Bt185 plasmid pBT1850055	CP014285.1	55,372	35.6	94	0	-	-	putative degenerated plasmid prophage
B. thuringiensis QZL38 plasmid p.4	CP032612.1	68,022	35.6	125	0	-	-	putative degenerated plasmid prophage

Table S11. General characteristics of Group 11 genomes.

Name	Accession Number	Genome size, bp	Number of protein-coding genes	Number of tRNAs and pseudo-tRNAs	Plasmid partitioning protens	Site-specific recombinases	Related bacteriophages (cover/iden) (length)	Type	References
B. cereus FORC087 plasmid pFORC087.3	CP029457.1	68,982	98	0	- ParA-Ib*<br>- RHH**<br>-RR***	- ResInv****	-	putative plasmid prophage	-
B. thuringiensis HD1011 plasmid 3	CP009332.1	82,340	105	0	- ParA-1b<br>- RHH	- ResInv	-	putative plasmid prophage	[1]
B. cereus PL1 plasmid pBwiPL1-2	AP022645.1	72,105	103	0	- ParA-Ib<br>- RHH<br>-RR	- ResInv	-	putative degenerated plasmid prophage	[2]
B. tropicus AOA-CPS1 plasmid pBClin15	CP049020.1	58,549	88	0	- ParA-Ib<br>- RHH<br>-RR	- ResInv	-	putative degenerated plasmid prophage	-
B. thuringiensis HD682 plasmid pBGN_2	CP009718.1	56,203	81	0	- ParA-Ib<br>- RHH	-	-	putative degenerated plasmid prophage	[1]
B. anthracis MCCC 1A01412 plasmid p1	CP031644.1	68,426	101	0	- ParA-Ib<br>- RHH	- ResInv	-	putative plasmid prophage	-
B. thuringiensis HD12 plasmid pHD120038	CP014849.1	38,333	73	1	- RR	- Xer*****	Bacillus phage vB_BthS-HD29phi (73/98) (32181)	putative degenerated plasmid prophage	-
B. weihenstephanensis KBAB4 plasmid pBWB404	CP000907.1	52,830	76	0	- ParA-Ib<br>- RHH<br>- RR	-	-	putative degenerated plasmid prophage	[3], [4]
B. thuringiensis serovar *tenebrionis* YBT-1765 plasmid pBMB165	CP002178.1	77,627	105	0	- ParA-Ib<br>- RHH<br><br>- ParM<br>- RHH	- 2 Xer	- vB_BtS_BMBtp3 (66/100) (51366)	proven plasmid-integrated prophage	[5]
B. thuringiensis HD12	CP014850.1	39,023	59	0	-	- ResInv	-	putative	-

plasmid pHD120039								degenerated plasmid prophage	
B. thuringiensis HD-771 plasmid p03	CP003755.1	69,876	84	0	- ParM	- 2 Xer<br>- ResInv	-	putative degenerated plasmid prophage	-
B. mycoides Gnyt1 plasmid unnamed6	CP020749.1	64,682	80	0	-ParM<br>-RHH	- 2 ResInv	-	putative degenerated plasmid prophage	-
B. thuringiensis MYBT18246 plasmid p46701	CP015358.1	46,701	61	1	- ParA-Ib<br>- RHH	- Xer	-	putative degenerated plasmid prophage	[6]
B. thuringiensis YBT-1518 plasmid pBMB0229	CP005936.1	45,206	61	1	- ParA-Ib<br>- RHH	-	-	putative plasmid prophage	[7]
B. thuringiensis CTC plasmid	CP013273.1	25,529	45	0	-	-	- Lactococcus phage p2<br>(89/94) (27595)<br>- Lactococcus phage jj50<br>(89/93) (27453)<br>- Lactococcus phage sk1<br>(89/93) (28451)	putative virulent phage	[8]
B. mycoides BPN401 plasmid pl50	CP031073.1	50,441	79	0	- ParM<br>- RHH	-	-	putative plasmid prophage	-
B. cereus NC7401 plasmid pNC1	AP007211.1	47,972	81	0	- ParM<br>- RHH	-	- Bacillus phage PfIS075<br>(98/100) (48709)<br>- Bacillus phage 250<br>(73/99) (56505)	proven plasmid prophage	[9], [10]
B. mycoides Gnyt1 plasmid unnamed7	CP020750.1	51,563	88	0	- ParA-Ib<br>- RHH	-	-	putative plasmid prophage	-
B. thuringiensis HD-771 plasmid p05	CP003757.1	45,262	72	0	- ParA-Ib<br>-RHH	- Xer	-	putative degenerated	-

								plasmid prophage	
B. cereus FORC021 plasmid	CP014487.1	41,600	74	0	- ParM<br>- RHH	-	-	putative plasmid prophage	[11]
B. cereus 03BB108 plasmid pBFI_5	CP009637.1	42,470	72	0	- ParM<br>- RHH	-	-	putative plasmid prophage	[1]
B. thuringiensis HS18-1 plasmid pHS18-5	CP012104.1	42,726	72	0	- ParA-Ib<br>- RHH	- ResInv	-	putative plasmid prophage	[12]
B. cereus Q1 plasmid pBc53	CP000229.1	52,766	75	0	- ParM<br>- RHH	- ResInv	-	putative plasmid prophage	[13]
B. thuringiensis MC28 plasmid pMC54	CP003689.1	54,484	78	1	- ParM<br>- RHH	- Xer	-	putative plasmid prophage	[14]
B. thuringiensis L-7601 plasmid unnamed1	CP020003.1	55,336	78	0	- ParM<br>- RHH	- Xer	-	putative plasmid prophage	[15]

Table S12. List of twenty-five singleton genomes. ParA-Ib – ParA Ib-type ATPase; RHH – ribbon-helix-helix domain-containing protein; RR – Replix_Relax superfamily protein; ResInv – site-specific serine recombinase, Resolvase and Invertase subfamily; Xer – Xer family site-specific tyrosine recombinase.

References

1. Johnson, S. L. *et al.* Finished genome sequence of *Bacillus cereus* strain 03bb87, a clinical isolate with *B. anthracis* virulence genes.*Genome Announc*. **3**, DOI: https://doi.org/10.1128/genomeA.01446-14 (2015).
2. Miyazaki, K., Hase, E. & Maruya, T. Complete genome sequence of *Bacillus cereus* strain pl1, isolated from soil in Japan.*Microbiol. Resour. Announc*.**9**, DOI: https://doi.org/10.1128/mra.00195-20 (2020).
3. Lapidus, A. *et al*. Extending the *Bacillus cereus* group genomics to putative food-borne pathogens of different toxicity.*Chem. Interactions* **171**, 236–249, DOI: https://doi.org/10.1016/j.cbi.2007.03.003 (2008).
4. Sorokin, A. *B. thuringiensis genetics and phages – from transduction and sequencing to recombineering.* (*B. thuringiensis Biotechnology. Springer*, 2012
5. Wang, Y. et al. Cloning and analysis of a large plasmid pbmb165 from *Bacillus thuringiensis* revealed a novel plasmid organization. *PLoS ONE* **8**, DOI: https://doi.org/10.1371/journal.pone.0081746 (2013).
6. Hollensteiner, J., *et al*. Complete Genome sequence of the nematicidal *Bacillus thuringiensis* MYBT18247. *J. Biotechnol.* **260,** 48-52, DOI: https://doi.org/10.1016/j.jbiotec.2017.09.003 (2017).
7. Wang, P, *et al*. Complete genome sequence of *B. thuringiensis* YBT-1518, a typical strain with high toxicity to nematodes. *J. Biotechnol.* **171**, 1-2, DOI: https://doi.org/10.1016/j.jbiotec.2013.11.023 (2014).
8. Dong, Z. *et al*. Complete genome sequence of *B. thuringiensis* CTC-A typical strain with high production of S-layer proteins. *J. Biotechnol.* **220**, 100-101, DOI: https://doi.org/10.1016/j.jbiotec.2015.12.027 (2016).
9. Takeno, A. *et al*. Complete genome sequence of *B. cereus* NC7401, which produces high levels of the emetic toxin cereulide. *J. Bacteriol.* **194**, 4767-4768, DOI: https://doi.org/10.1128/JB.01015-12 (2012).
10. Geng, P., *et al*. Identification and genomic comparison of temperate bacteriophages derived from emetic *B. cereus*. *PloS one* **12**, e0184572, DOI: https://doi.org/10.1371/journal.pone.0184572 (2017).
11. Chung, H. *et al*. Genome Sequence of *B. cereus* FORC_021, a Food-Borne Pathogen Isolated from a Knife at a Sashimi Restaurant. *J. Microbiol. Biotechnol.* **26**, 2030-2035, DOI: https://doi.org/10.4014/jmb.1604.04094 (2016).
12. Li, Qiao, *et al*. Complete genome sequence of *B. thuringiensis* HS18-1. *J. Biotechnol* **214**, 61-62, DOI: https://doi.org/10.1016/j.jbiotec.2015.08.017 (2015).
13. Xiong, Z. *et al*. Complete genome sequence of the extremophilic *B. cereus* strain Q1 with industrial applications. *J. Biotechnol.* **191**, 1120-1121 DOI: https://doi.org/10.1128/JB.01629-08 (2009).
14. Guan, Peng, *et al*. Complete genome sequence of *B. thuringiensis* serovar Sichuansis strain MC28. *J. Biotechnol.* **194**, 6975-6975 DOI: https://doi.org/10.1128/JB.01861-12 (2012).
15. Cao, Z. *et al*. Complete genome sequence of *B. thuringiensis* L-7601, a wild strain with high production of melanin. *J. Biotechnol.* **275**, 40-43, DOI: https://doi.org/10.1016/j.jbiotec.2018.03.020 (2018).

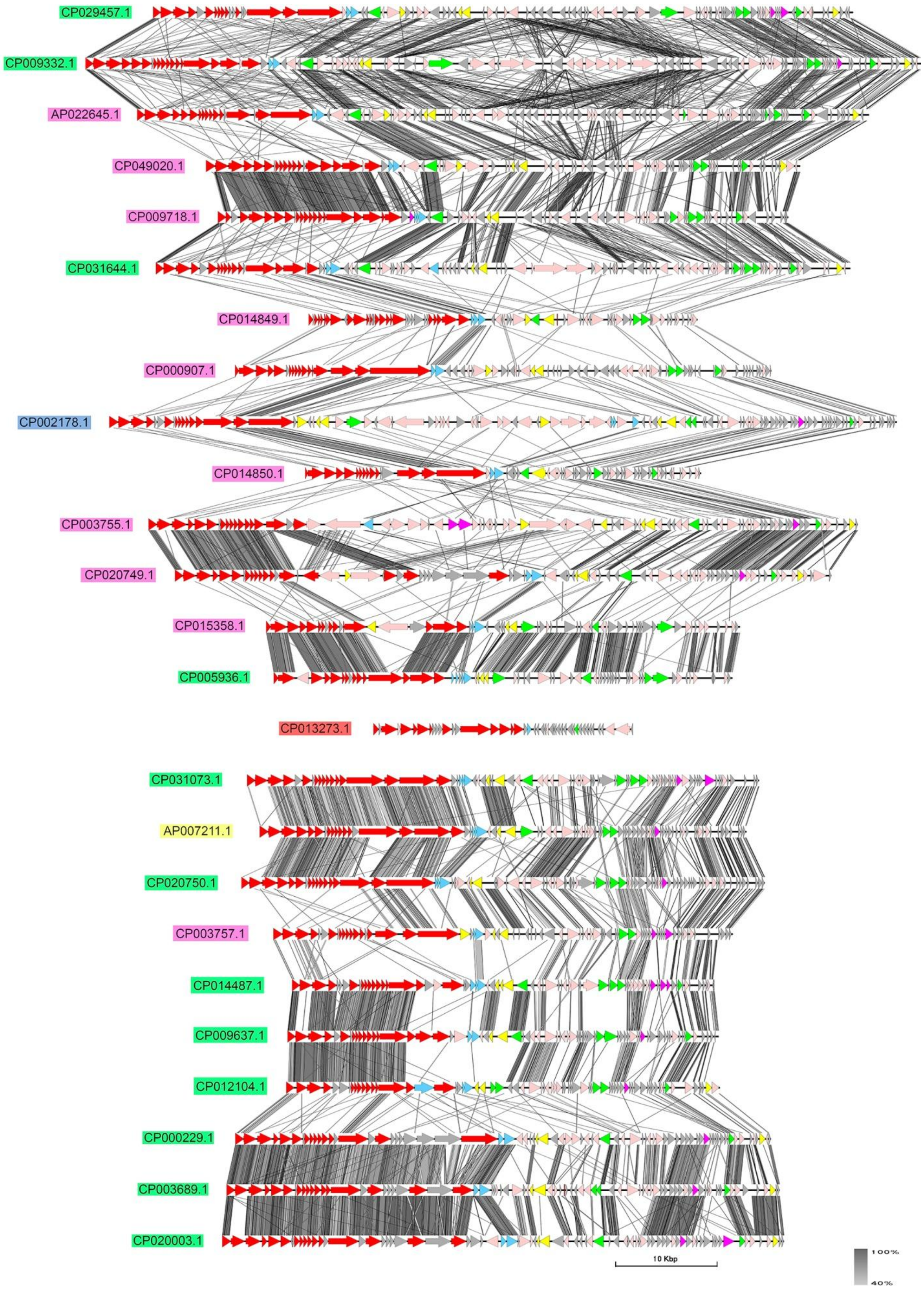

Figure S1. The TBLASTX genome comparison of plasmids not assigned to any group (singletons), performed and visualized with Easyfig 2.2.2. (https://mjsull.github.io/Easyfig/). GenBank accession numbers of putative plasmid prophages are highlihted in green, putative degenerated plasmid prophages – pink, putative virulent phage – red, proven plasmid-integrated prophage – blue, proven plasmid prophage – yellow. The gray regions between the genome maps indicate the level of identity from 40% to 100% (see the legend on the right).
